# Large-scale kinetic metabolic models of *Pseudomonas putida* KT2440 for consistent design of metabolic engineering strategies

**DOI:** 10.1186/s13068-020-1665-7

**Published:** 2020-02-28

**Authors:** Milenko Tokic, Vassily Hatzimanikatis, Ljubisa Miskovic

**Affiliations:** Laboratory of Computational Systems Biotechnology (LCSB), École Polytechnique Fédérale de Lausanne (EPFL), 1015 Lausanne, Switzerland

**Keywords:** *Pseudomonas putida*, Large-scale and genome-scale kinetic models, Nonlinearity, Metabolism, Thermodynamics, Kinetic parameters, Uncertainty, Metabolic engineering, Stress conditions

## Abstract

**Background:**

*Pseudomonas putida* is a promising candidate for the industrial production of biofuels and biochemicals because of its high tolerance to toxic compounds and its ability to grow on a wide variety of substrates. Engineering this organism for improved performances and predicting metabolic responses upon genetic perturbations requires reliable descriptions of its metabolism in the form of stoichiometric and kinetic models.

**Results:**

In this work, we developed kinetic models of *P. putida* to predict the metabolic phenotypes and design metabolic engineering interventions for the production of biochemicals. The developed kinetic models contain 775 reactions and 245 metabolites. Furthermore, we introduce here a novel set of constraints within thermodynamics-based flux analysis that allow for considering concentrations of metabolites that exist in several compartments as separate entities. We started by a gap-filling and thermodynamic curation of iJN1411, the genome-scale model of *P. putida* KT2440. We then systematically reduced the curated iJN1411 model, and we created three core stoichiometric models of different complexity that describe the central carbon metabolism of *P. putida*. Using the medium complexity core model as a scaffold, we generated populations of large-scale kinetic models for two studies. In the first study, the developed kinetic models successfully captured the experimentally observed metabolic responses to several single-gene knockouts of a wild-type strain of *P. putida* KT2440 growing on glucose. In the second study, we used the developed models to propose metabolic engineering interventions for improved robustness of this organism to the stress condition of increased ATP demand.

**Conclusions:**

The study demonstrates the potential and predictive capabilities of the kinetic models that allow for rational design and optimization of recombinant *P. putida* strains for improved production of biofuels and biochemicals. The curated genome-scale model of *P. putida* together with the developed large-scale stoichiometric and kinetic models represents a significant resource for researchers in industry and academia.

## Background

*Pseudomonas putida* recently emerged as one of the most promising production hosts for a wide range of chemicals, due to its fast growth with a low nutrient [1] and cellular energy [2] demand, considerable metabolic versatility [3], ability to grow in wide range of chemicals [4, 5], suitability for genetic manipulations [6], and its robustness and high flexibility to adapt and counteract different stresses [7]. One of the main advantages of *P. putida* compared to heavily used industrial workhorses like *E. coli* is its superior tolerance to toxic compounds such as benzene, toluene, ethylbenzene, xylene, *n*-hexane and cyclohexane [8, 9]. For example, Ruhl et al. in 2009 showed that some *P. putida* strains such as DOT-T1E, S12, and VLB120 are able to grow in high concentrations of *n*-butanol [5] up to 6% (vol/vol), whereas the concentrations of 1.5% (vol/vol) cause significant growth decrease in *E. coli* [8].

Recent efforts toward understanding and improving the behavior and systemic properties of *P. putida* metabolism resulted in several genome-scale reconstructions. The first reconstructed Genome-Scale Model (GEM) of *P. putida* KT2440, iJN746, was published in 2008 and it comprised 911 metabolites, 950 reactions, and 746 genes [10]. It was rapidly followed by the publication of iJP815 [11] and other reconstructions [12, 13]. The inconsistencies among these models motivated Yuan et al. in 2017 to build so-called pathway-consensus model PpuQY1140 [14]. The so far most complete GEM of *P. putida* KT2440, iJN1411, was reconstructed in 2017 by Nogales et al. [15], and it contains 2057 metabolites, 2581 reactions, and 1411 genes. The GEMs have been used for studying metabolic features of *P. putida* including the enhanced production of poly-hydroxyalkanoates [16], reconciliation of key biological parameters for growth on glucose under carbon-limited conditions [17], and identification of essential genes for growth on minimal medium [18]. However, stoichiometric models cannot be used to describe the dynamic metabolic responses to changes in cellular and process parameters nor they can consider regulation at the enzyme and post-translational level [19]. Therefore, kinetic metabolic models are needed to address these requirements.

Multiple small-scale kinetic models of *P. putida* metabolism were developed to model the growth and changes in extracellular concentrations [20–29]. Bandyopadhyay et al. in 1998 used a simple Monod model to study the effect of phenol degradation in *P. putida* MTCC 1194 [22]. Wang and Loh in 2001 modeled the co-metabolism of phenol and 4-chlorophenol in the presence of sodium glutamate in *P. putida* ATCC 49451 [29], and their kinetic model accounted for cell growth, the toxicity of 4-chlorophenol, and cross-inhibitions among the three substrates. Other models were used for studying growth during benzene [20], toluene [20, 24–26, 28] and phenol biodegradation [20], growth and biosynthesis of medium-chain-length poly-(3-hydroxyalkanoates) [21] and dibenzothiophene desulfurization [23, 27].

More recently, Sudarsan et al. in 2016 developed a kinetic model of the β-ketoadipate pathway in *P. putida* KT2440 that contained mass balance equations for both extracellular and intracellular metabolites described by mechanistic rate expressions based on in vitro investigation of the participating enzymes [30]. Chavarria et al. in 2016 modeled the dynamics of fructose uptake in *P. putida* KT2440 while taking into account the dynamics of gene expression, protein stability, enzymatic activity and the concentrations of intracellular and extracellular metabolites [31].

All these kinetic models are of limited size and with ad hoc stoichiometry, i.e., their stoichiometry was built for a specific purpose and without justifications how their metabolites and reactions were chosen [32, 33]. Therefore, a need for developing large-scale kinetic models capable of reliably identifying metabolic engineering targets for production of the desired chemicals exist [19]. However, construction of large-scale kinetic models remains a challenging task. Each reaction in a kinetic model requires a matching kinetic rate expression along with values of kinetic parameters, which are frequently unknown. Moreover, even if the values of kinetic parameters are available in the literature and databases, their reported values are often spanning several orders of magnitude. Additionally, partial experimental fluxomic and metabolomic data and estimation errors in related thermodynamic properties [19] hinder determining unique steady-state metabolic fluxes and metabolite concentrations [34]. As a consequence, there is no unique model capable of describing the observed physiology. Instead, to overcome this issue, a population of kinetic models is constructed, and statistical methods are used to analyze and predict the metabolic responses in the system [19, 34].

In this work, we first performed a thermodynamic curation of the iJN1411 GEM, i.e., we estimated the standard Gibbs energy of formation of metabolites, adjusted these values for pH and ionic strength in the studied physiological condition, and used these values together with the concentrations of metabolites to calculate the transformed Gibbs free energy of reactions [35–40]. We then performed the gap-filling of iJN1411 and systematically reduced this model to derive three different-complexity core models of *P. putida* central carbon metabolism. We provide the models of three different sizes to allow modelers to make a trade-off between the accuracy of the models and the model complexity. The level of details of important metabolic interactions described in the model affects the model accuracy. The more detailed model, the better is its accuracy. However, as the model complexity increases, the portion of available data of intracellular metabolite concentration and metabolic flux is rapidly decreasing, i.e., uncertainty in the system is increasing [19]. Next, we applied ORACLE [34, 41–50], a computational framework based on Monte Carlo sampling, to construct large-scale kinetic metabolic models of *P. putida* KT2440. The potential of developed kinetic models for the design of improved production strains of *P. putida* was demonstrated through two studies: (i) predicting metabolic responses of a wild-type *P. putida* strain to single-gene knockouts; and (ii) improving the responses of this organism to the stress conditions of increased ATP demand.

## Results and discussion

### Thermodynamically curated genome-scale model of *P. putida*

#### Integration of thermodynamics data

Methods that use thermodynamic data such as the thermodynamics-based flux analysis TFA [35–39] allow to: (i) integrate the metabolomics and fluxomics data into models, and compute values of metabolic fluxes and metabolite concentrations whose experimental measurements are not available; (ii) eliminate in silico designed biosynthetic pathways not obeying the second law of thermodynamics [51, 52]; (iii) eliminate infeasible thermodynamic cycles [53–55]; and (iv) identify how far reactions operate from thermodynamic equilibrium [46, 56]. Despite the fact that usefulness of thermodynamics has been demonstrated in many applications, only a few reconstructed GEMs are curated for this important property [46, 57–60].

We used Group Contribution Method (GCM) [61, 62] to assign the standard Gibbs free energy of formation to 62.3% metabolites and the standard Gibbs free energy of reaction to 59.3% reactions from the iJN1411 model. We calculated the standard Gibbs free energies for all metabolites and reactions participating in the pathways of central carbon metabolism (glycolysis, gluconeogenesis, pentose phosphate pathway, tricarboxylic acid (TCA) cycle). In contrast, we could estimate the standard Gibbs free energy of reaction for only 3.3% reactions in the poly-hydroxyalkanoates (PHA) metabolism because the majority of involved metabolites from these pathways have the structures with unknown residuals which precluded computation of the thermodynamic properties.

#### Integration of physiology data and gap-filling

We integrated experimental measurements of glucose uptake and biomass yield on glucose [63] and metabolite concentrations [64] into the thermodynamically curated model iJN1411. The performed TFA indicated that the model predicted ranges of ATP concentrations (Additional file 1: Table S9) could not match the values reported in the literature [64, 65]. A reason for this mismatch could lie in the fact that the H+/ATP stoichiometry in the electron transport chain (ETC) of *P. putida* might be inaccurately determined in iJN1411 which would lead to large discrepancies in ATP yield on glucose [3, 66]. Here, we investigated another venue and hypothesized that iJN1411 is missing a critical reaction in the ATP-related metabolism. Therefore, to make model predictions consistent with the experimental observations, we used the gap-filling procedure proposed by Chiappino-Pepe et al. in 2017 [60] and later used by Hadadi et al. in 2019 [67]. The gap-filling procedure is metabolic-task-driven [68, 69], where a metabolic task such as the production of a biomass precursor is defined and mixed-integer linear programming (MILP) is used to identify a minimal number of gap-filling reactions required to perform the task. The candidate reactions for gap-filling can be taken from: (i) databases such as KEGG [70], MetaCyc [71], and Atlas of Biochemistry [72]; (ii) genome-scale models of similar organisms; or (iii) an ad hoc set of reactions chosen by experts. Here, we defined a metabolic task of matching experimentally measured values of glucose uptake, specific growth rate, and ATP concentration (“Methods”). The set of candidate reactions was taken from iJO1366 GEM of *E. coli*, a well-studied species of Gram-negative rod-shaped bacteria [73]. The solution of the MILP problem indicated that one reaction, sulfate adenyltransferase (SADT2), is missing in the iJN1411. SADT2 plays a role in cysteine formation, and similarly to sulfate adenylyltransferase (SADT), which already exists in the iJN1411, it catalyzes the production of cysteine precursor adenosine 5′-phosphosulfate from ATP and SO_4_. The production of adenosine 5′-phosphosulfate catalyzed by SADT2 is coupled with GTP consumption, whereas this coupling is absent in SADT. Since the experimental evidence supports that GTP hydrolysis enhances the rate of adenosine 5′-phosphosulfate formation [74], we included this reaction into iJN1411. The thermodynamically curated, gap-filled, model iJN1411 was consistent with the experimental values of both fluxomics and metabolomics data. Interestingly, when we replaced SADT2 with SADT in iJO1366 (*E. coli*), the iJO1366 could not predict experimentally measured values of ATP in *E. coli* [75].

### Core reduced stoichiometric models of *P. putida*

#### Reconstruction of core reduced models

Using as a basis the curated iJN1411, we employed the redGEM [76] and lumpGEM [77] algorithms to construct a family of three core reduced stoichiometric models of *P. putida* of different complexity. The reduced models were constructed in two steps.

**Fig. 1:**
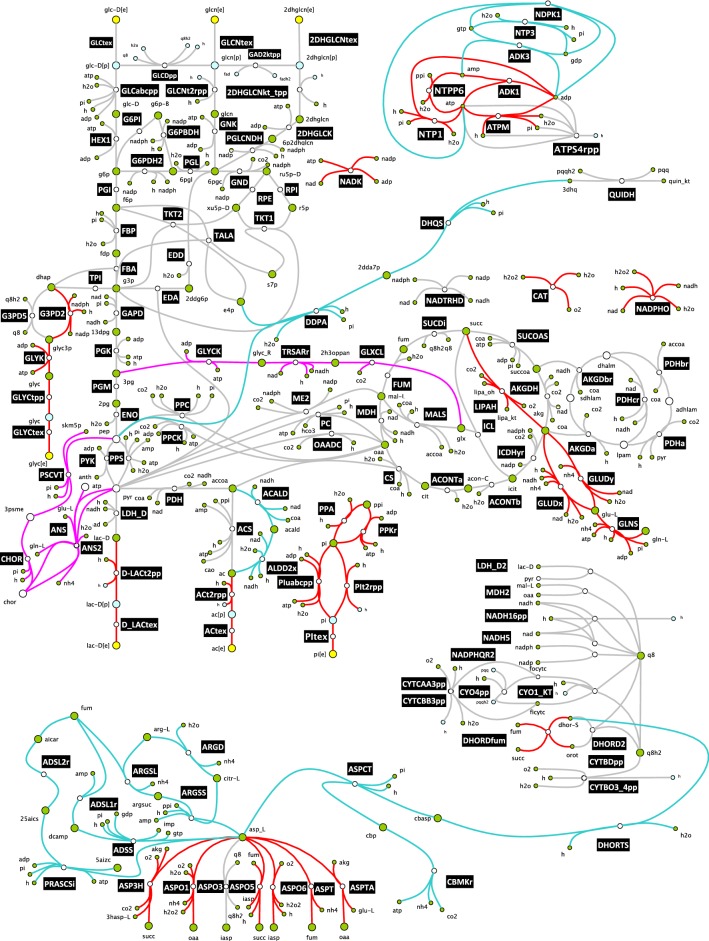
The core networks generated by the redGEM algorithm from iJN1411 genome-scale model. The core network was built around reactions (grey) that belong to the six subsystems of central carbon metabolism (glycolysis and gluconeogenesis, pentose phosphate pathway, pyruvate metabolism, TCA cycle and oxidative phosphorylation). Reactions belonging to one-reaction step, two-reaction-step, and three-reaction-step pairwise connections between the six subsystems are marked in red, cyan and magenta, respectively. The stoichiometry of the reduced models and a complete list of reactions and metabolites are provided in Additional file 9: File S2, Additional file 10: File S3 and Additional file 13: File S1

In the first step, the redGEM algorithm produced core networks focused around six central carbon subsystems of iJN1411: glycolysis and gluconeogenesis, pentose phosphate pathway, pyruvate metabolism, TCA cycle and oxidative phosphorylation (Fig. 1). The core networks of the three reduced models differed in the size depending on the number reactions in the pairwise interconnections between the subsystems (“Methods”). In the smallest core network, the D1 core network, the subsystems were pairwise interconnected by up to one reaction. In the D2 and D3 core networks, the subsystems were pairwise interconnected by up to two and three reactions, respectively. The D1, D2, and D3 core networks contained 278, 307, and 343 reactions, and 286, 306, and 336 metabolites, respectively (Table 1).

**Table 1:** Three reduced core models D1, D2 and D3

	D1	D2	D3
Reactions	828	704	750
Core	278	307	343
Lumped	550	397	407
% of reactions with estimated standard Gibbs free energy	70.4	70.8	62.3
Metabolites	286	306	336
Cytosolic	156	174	200
Periplasmic	70	71	74
Extracellular	60	61	62
% of metabolites with estimated standard Gibbs free energy	81.8	82.7	82.1

In the second step, the lumpGEM algorithm was used to connect the metabolites of the three core networks with 102 biomass building blocks (BBB) of the iJN1411 biomass reaction (Methods). lumpGEM generates lumped reactions that account for the production of BBBs from the metabolites of the core metabolic networks, i.e., it allows for modeling the fate of all metabolites along the synthesis routes and quantifying the cost of all precursor metabolites and cofactors [77]. Moreover, it allows capturing the flexibility in the metabolic network of *P. putida* by generating alternative lumped reactions towards BBBs. The lumpGEM appended to the D1, D2, and D3 core networks 550, 397, and 407 lumped reactions, respectively (Table 1).

The resulting D1 model contained 828 reactions and 286 metabolites distributed over cytosol, periplasm and the extracellular space (Table 1). For 583 out of 828 (70.4%) reactions and 234 out of 286 (81.8%) metabolites from D1 we could calculate the thermodynamic properties (Table 1). The D2 model contained 704 reactions and 306 metabolites. Out of these, we could calculate the thermodynamic properties for 498 (70.8%) reactions and 253 (82.7%) metabolites. The D3 model had a total of 750 reactions and 336 metabolites with calculated thermodynamic properties for 467 (62.3%) reactions and 276 (82.1%) metabolites (Table 1).

We performed the consistency checks of D1, D2, and D3 against their genome-scale counterpart iJN1411 according to the procedure proposed in Ataman et al. [76], and we found that they were consistent with the GEM in terms of biomass yields, gene essentiality, and flux and concentration variability (“Methods”).

#### Essentiality of genes encoding for EDA and EDD

The Entner–Doudoroff (ED) pathway is essential for the growth of *P. putida* on glucose, which is experimentally confirmed by the absence of the growth in mutants lacking the key enzymes 2-dehydro-3-deoxy-phosphogluconate aldolase (EDA) and 6-phosphogluconate dehydratase (EDD) [63, 78, 79]. Using TFA, we found that these genes are not essential on glucose minimal medium (Additional file 1: Table S7) in D2 and iJN1411 because these models can replenish the pool of triose phosphates through the pentose phosphate pathway. Interestingly, Nogales et al. in 2017 have used the minimization of metabolic adjustment (MOMA) method [80] and found that EDA and EDD are essential on glucose minimal medium in iJN1411 [15]. The previous GEMs of *P. putida* were not able to predict the essentiality of these genes [18].

**Fig. 2:**
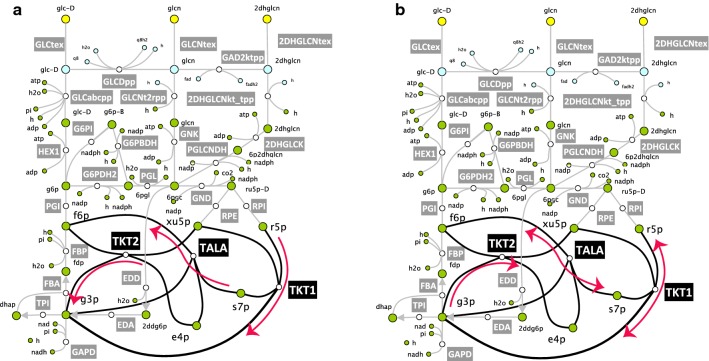
The directionality of transketolase 2 (TKT2) impacts the in silico essentiality of two genes encoding EDD and EDA from the Entner–Doudoroff pathway. **a** If TKT2 operates towards production of g3p, then due to the stoichiometric coupling transketolase 1 (TKT1) and transaldolase (TALA) are unidirectional and EDD and EDA are not in silico essential. **b** If TKT2 operates towards consumption of g3p, EDD and EDA are in silico essential irrespectively of the directionalities of TKT1 and TALA

We analyzed how the directionalities of reactions from the pentose phosphate pathway impact the essentiality of EDA and EDD in D2. We found that the directionalities of three reactions that have glyceraldehyde 3-phosphate (g3p) as reactant (transaldolase, TALA, and two transketolases, TKT1 and TKT2) determine if EDD and EDA are in silico essential. When directionality of TKT2 was imposed towards production of g3p, TALA and TKT1 became exclusively unidirectional towards consumption of g3p and production of g3p, respectfully (Fig. 2a), and EDA and EDD were not essential. In contrast, when TKT2 operated towards consumption of g3p EDA and EDD were essential regardless the directionality of the other two reactions (Fig. 2b). Therefore, to ensure the consistency of in silico and experimentally observed gene essentiality of EDD and EDA in the subsequent studies we imposed the directionality of TKT2 towards consumption of g3p.

### Kinetic study of wild-type *P. putida* physiology

#### Model responses to six single-gene knockouts

The reduced D2 model was used as a scaffold for constructing a population of thermodynamically feasible kinetic models. We preconfigured this model for kinetic studies (“Methods”) and we performed TFA with a novel set of constraints that allow for considering concentrations of metabolites across several compartments to integrate 57 experimentally measured intracellular metabolite concentrations [64] (“Methods”). We found that all reaction directionalities within the obtained thermodynamically feasible steady-state flux and metabolite concentration profile were in agreement with the pre-assigned directionalities from iJN1411 [15] (Additional file 1: Table S1).

We used ORACLE [34, 41–50] to construct a population of 50,000 nonlinear kinetic models around the computed steady-state flux and concentration profile (“Methods”). The constructed models contained the experimental values for 21 Michaelis constants (*K*_m_’s) available for the *Pseudomonas* genus in the Brenda database [81–84]. The resulting structure of kinetic models consisted of 775 enzymatic reactions and 245 mass balances for metabolites distributed over cytosol and periplasm.

**Fig. 3:**
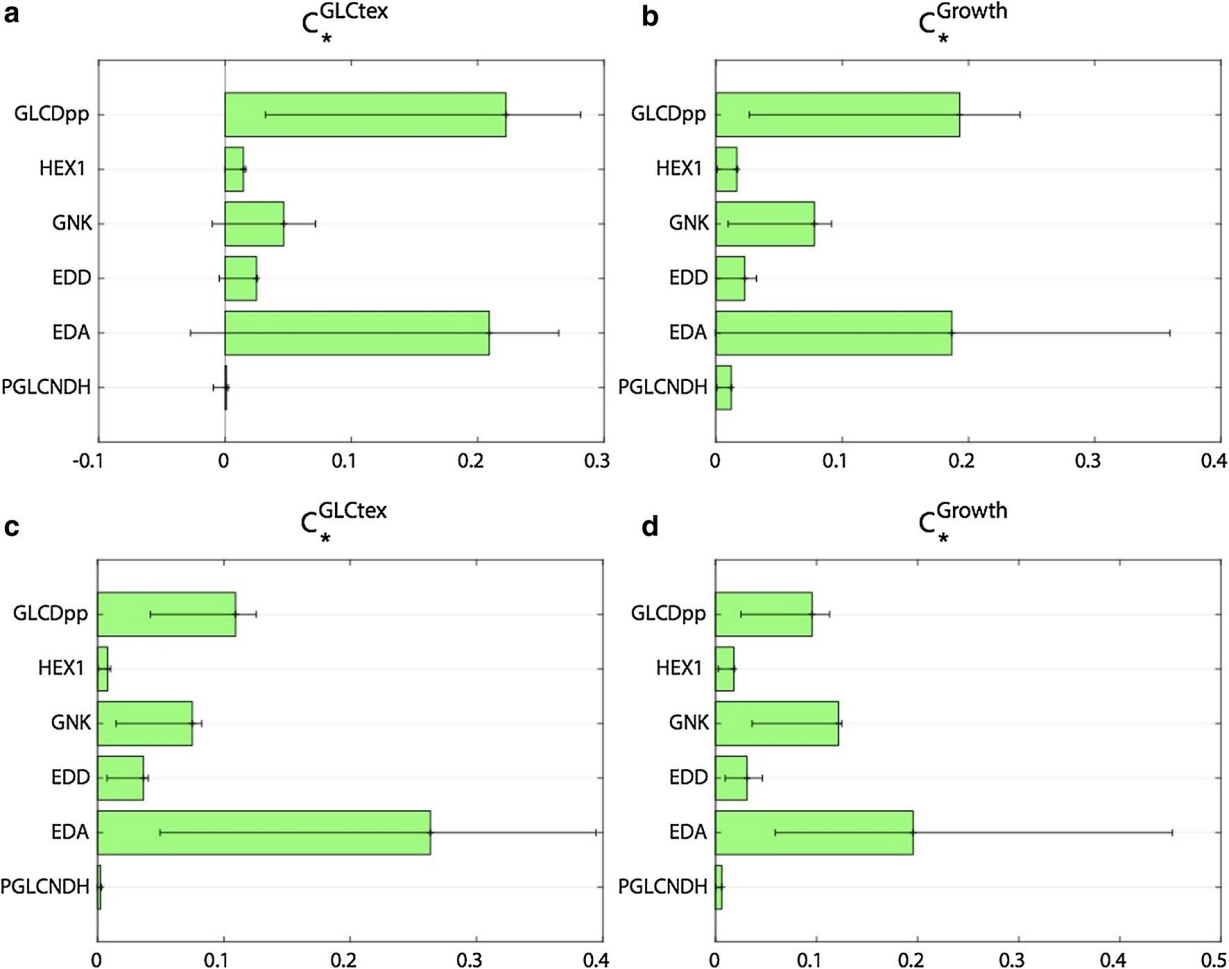
Distribution of the control coefficients of glucose uptake (GLCtex) and specific growth rate (growth) for the wild-type physiology of *P. putida*. The control coefficients of glucose uptake (**a**) and specific growth rate (**b**) were first computed using an unbiased sampling in ORACLE, and then further refined using the machine learning methodology iSCHRUNK (**c**, **d**). The green bars represent the mean values of the control coefficients, whereas the error bars correspond to the 25 and 75 percentiles of the distributions

To evaluate the predictive capabilities of the constructed models, we computed the flux control coefficients of the intracellular fluxes in the metabolic network. The flux control coefficients quantify the relative steady-state change in fluxes in response to relative changes in parameters, and allow us to identify how control of the carbon and energy flows within the metabolic networks is redistributed [43, 85, 86]. We compared the flux control coefficients of glucose uptake and specific growth rate with respect to six enzymes (glucose dehydrogenase, GLCDpp, hexokinase, HEX1, gluconokinase, GNK, EDA, EDD, and phosphogluconate 2-dehydrogenase, PGLCNDH) with the experimentally measured responses of the glucose uptake and specific growth rate to single-gene knockouts of these six enzymes [63]. The computed control coefficients for the glucose uptake and specific growth rate were in a qualitative agreement with the data reported by del Castillo et al. [63] (Additional file 1: Table S2), i.e., a decrease in the enzyme activity of the six enzymes would lead to a decrease in both the glucose uptake and specific growth rate (Fig. 3a, b). In contrast, the results of in silico gene knockouts performed with FBA and TFA on iJN1411 and D2 have shown no reduction in growth for four knockouts, ∆GLCDpp, ∆HEX1, ∆GNK, and ∆PGLCNDH (Additional file 1: Table S2). For ∆EDD and ∆EDA knockouts, iJN1411 and D2 with bidirectional TKT2 have shown a moderate decrease in growth, whereas, as discussed previously, D2 with imposed TKT2 directionality has correctly predicted the growth arrest for ∆EDD and ∆EDA knockouts.

A closer inspection of the flux control coefficients of glucose uptake revealed that for four enzymes (GNK, EDD, EDA and PGLCNDH) the error bars were spread around zero values (Fig. 3a). This meant that there was a subpopulation of models with inconsistent predictions with some of the six knockouts. In fact, only 4999 (~ 10%) out of 50,000 computed models were able to correctly predict responses to all six knockouts reported in del Castillo et al. [63] due to the large uncertainty in the assigned values of the kinetic parameters. This type of uncertainty is common in biochemical systems and recently proposed computational method iSCHRUNK allows to investigate and reduce the uncertainty, and therefore, to improve the predictive strength of kinetic models [19, 87, 88].

#### Refinement of model responses to six single-gene knockouts

We used iSCHRUNK to eliminate the inconsistencies with the experimental data observed for some of the predicted responses (“Methods”). The method allowed identifying seven kinetic parameters and their ranges that ensure the consistency of model responses with the experimental observations, and interestingly, all parameters were related to the ED pathway (Table 2).

**Table 2:** Ranges of the original set of parameters computed by ORACLE and of the refined set of parameters inferred by the iSCHRUNK method

Parameter	Original ranges (mM)	Refined ranges (mM)
$K_{{{\text{m}},2{\text{dhglcn}}}}^{{2{\text{DHGLCNtex}}}}$	6.83 ·× 10^−5^–0.68	6.83 ·× 10^−5^–2.34 · 10^−3^
$K_{{{\text{m}},2{\text{dhglcn}}}}^{{{\text{GAD}}2{\text{ktpp}}}}$	6.83 ·× 10^−5^–0.68	6.83 ·× 10^−5^–0.133
$K_{{{\text{m}},{\text{q}}8}}^{\text{GLCDpp}}$	3.81 ·× 10^−3^–37.71	3.81 ·× 10^−3^–0.899
$K_{{{\text{m}},{\text{glcn}}}}^{\text{GLCDpp}}$	0.01–107.37	0.01–5.76
$K_{{{\text{m}},{\text{glcn}}}}^{{{\text{GLCNt}}2{\text{rpp}}}}$	2.63 ·× 10^−5^–0.26	6.54 ·× 10^−4^–9.49 · 10^−4^
$K_{{{\text{m}},{\text{adp}}}}^{\text{GNK}}$	2.03 ·× 10^−3^–20	3.84 ·× 10^−2^–20
$K_{{{\text{m}},6{\text{pgc}}}}^{\text{GNK}}$	4.26 ·× 10^−5^–0.42	4.26 ·× 10^−5^–8.37 · 10^−2^

2DHGLCNtex, ketogluconate transport via diffusion extracellular to periplasm; GAD2ktpp, gluconate 2 dehydrogenase periplasm; GLCDpp, glucose dehydrogenase ubiquinone 8 as acceptor periplasm; GLCNt2rpp, d-gluconate transport via proton symport reversible periplasm; GNK, gluconokinase; 2dhglcn, 2-dehydro-d-gluconate; 6pgc, 6-phospho-d-gluconate; adp, ADP; glcn, d-gluconate; q8, ubiquinone-8

We generated a novel population of kinetic models with ORACLE with constrained ranges of these seven parameters as defined by iSCHRUNK, and with integrated experimental values for 21 km’s from the Brenda database, and we then computed the distributions of corresponding control coefficients for the glucose uptake and specific growth rate. Out of 50,000 models, 29,979 (~ 60%) models correctly predicted the changes in the glucose uptake rate to six single-gene knockouts [63] (Fig. 3c), while 35,955 (~ 72%) models agreed with the experimental data for the specific growth rate (Fig. 3d). In total, 26,120 (~ 52%) models were consistent with both the experimental data for the glucose uptake and the specific growth rate.

We discovered with iSCHRUNK that operating regimes of only a few enzymes determine metabolic responses to multiple single-gene knockouts. This emphasizes the significance of accurately determining the kinetic parameters of such important enzymes in order to obtain model responses consistent with the experimental observations. It will also be interesting to consider complex kinetic phenomena such as crowding when modeling kinetic properties of certain enzymes [89].

#### Assessment of estimated kinetic parameters

**Fig. 4:**
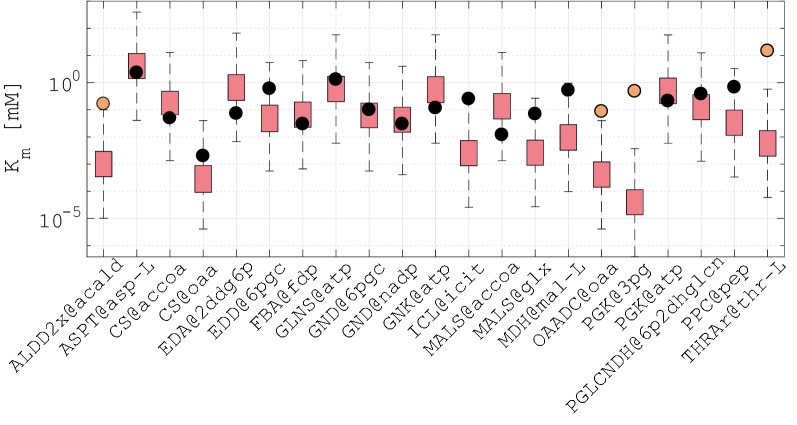
Estimates of Michaelis constants, *K*_m_’s, predicted by ORACLE. Distribution of *K*_m_’s estimated with ORACLE (red boxplots) without imposing experimental values from BRENDA (black circles denote experimental values of *K*_m_’s with consistent ORACLE estimates, whereas orange circles denote the ones with inconsistent ORACLE estimates). Whiskers represent minimal and maximal value predicted by ORACLE. Notation, e.g., PPC@pep denotes the Michaelis constant, i.e., the concentration of Phosphoenolpyruvate (pep) at which the reaction rate of Phosphoenolpyruvate carboxylase (PPC) is half of *V*_max_. Full names of reactions are provided in Additional file 1: Table S3

To obtain an unbiased assessment of the accuracy of our estimates, we computed a novel population of 50,000 models without imposing the experimentally available values of *K*_m_’s from the BRENDA database [81–84]. Comparison of our estimates against available values of *K*_m_’s from BRENDA showed that ORACLE could capture the ranges for 17 out of 21 km’s (Fig. 4). Considering that in the estimation process we did not use any kinetic parameters values and that the underlying system is underdetermined, this result is remarkable because it indicates that ORACLE with integrated fluxomics and metabolomics data together with the physico-chemical laws is capable to provide consistent estimates for a large number of kinetic parameters. This further suggests that ORACLE estimates can be used as hypothetic values for studies where the unknown kinetic parameters are required.

For the four remaining parameters such as Michaelis constant of l-threonine in Threonine aldolase or oxaloacetate in Oxaloacetate decarboxylase, ORACLE underestimated experimental values up to one and half orders of magnitude (Fig. 4). The discrepancies between the estimated and measured values of these parameters can originate from different sources: (i) the *K*_m_ values from BRENDA were measured on several different species from the *Pseudomonas* genus, whereas our *K*_m_ values were estimated using a *P. putida* model and the experimental data were acquired on *P. putida* (fluxomics data) and *P. taiwanensis* (metabolomics data); and (ii) large uncertainty in available and partially available experimental data. In general, the more experimentally measured data are available for integration in the models by ORACLE, the better their predictive capability will be.

### Kinetic study of increased ATP demand in *P. putida*

The robustness of microorganisms to environmental stresses encountered in industrial processes is a significant factor for choosing hosts for the production of biofuels and biochemicals. While stress-specific responses differ between various stresses such as product toxicity, heat, or osmotic stress, and different organisms can have different mechanisms for stress adaptation, counteracting stress requires energy [90]. For example, it was observed that a common factor in responses of *S. cerevisiae* to high ethanol concentration, osmotic stress, and high temperature is an increased demand for ATP [91]. The active removal of toxic compounds by energy-driven efflux pumps also significantly increases the energy demand in cells [7].

**Fig. 5:**
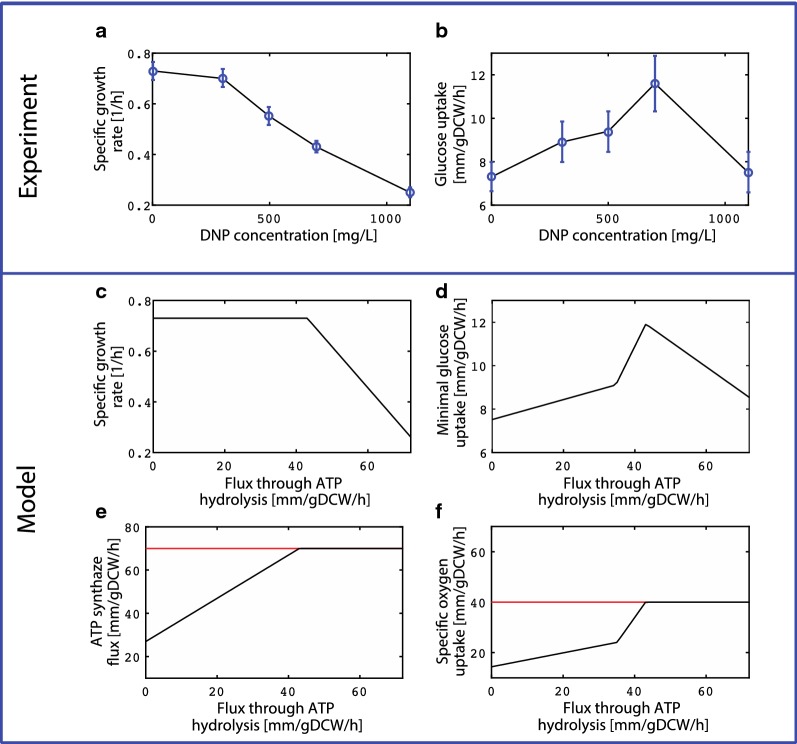
Fermentation profile of *P. putida* metabolism under increased ATP demand. Experimentally measured specific growth rate (**a**) and glucose uptake rate (**b**) of *P. putida* as the ATP demand induced by titration of 2,4 dinitrophenol (DNP) increases. The profiles of specific growth rate (**c**), glucose uptake rate (**d**), flux through ATP synthase (**e**) and oxygen uptake rate (**f**) computed by TFA using the reduced D2 model. The identical (**c**–**f**) profiles were obtained when iJN1411 was used in TFA, which further demonstrates the consistency of the reduced D2 model with iJN1411

Ebert and co-workers investigated how increased ATP demand affects the *P. putida* metabolism by titrating 2,4-dinitrophenol (DNP), and they demonstrated that DNP concentrations below 300 mg/l did not impact the specific growth rate of *P. putida* [7]. Above the concentration of 300 mg/l, DNP caused a significant reduction of *P. putida*’s specific growth rate and increase of the glucose uptake (Fig. 5a, b). At the concentration of 700 mg/l of DNP, glucose uptake reached the maximum of ~ 11 mmol/gDCW/h. For larger values of DNP concentration, both the glucose uptake and the specific growth rate declined.

In comparison, *E. coli* shows a significant reduction in the specific growth rate already at the concentrations of 138 mg/l [92]. It is argued in the literature that, compared to *E. coli*, *P. putida* superior capability of counteracting different types of stress and in particular oxidative stress stems from the specific metabolic arrangement of its glycolysis [65, 78, 93]. *P. putida* catabolizes glucose predominantly through Entner–Doudoroff pathway, whereas the Embden–Meyerhof–Parnas (EMP) pathway operates in a gluconeogenic fashion [78]. In contrast, *E. coli* has functional both EMP and ED pathway. However, the glucose metabolism in this organism is carried out through the EMP pathway, while the ED pathway remains mostly inactive. Hollinshead et al. in 2016 showed that about 90% of flux in *E. coli* is channelled through EMP pathway while the flux through ED pathway was negligible [94]. The active ED pathway allows to *P. putida* to generate NAPDH which is required to counteract environmental stresses [65, 78].

We undertook to investigate does the biochemical network of *P. putida* has the potential to produce enough ATP to cope with the stress. To this aim, we first used our stoichiometric model to assess the stoichiometric capacity of this organism to produce ATP, and then, we used the developed kinetic model to identify metabolic engineering strategies to steer the system towards attaining that capacity.

#### Assessing the stoichiometric capacity of *P. putida* for ATP production

We preconfigured the model for this study (Methods) and used it to simulate the impact of increased ATP demand on the *P. putida* metabolism by gradually increasing the minimally required flux through ATP hydrolysis in increments of 1 mmol/gDCW/h (Fig. 5). We set the upper bound of the specific growth rate to 0.73 1/h, as reported in Ebert et al. [7] for the DNP concentration of 0 mg/l. Based on the performed sensitivity analysis of model responses to upper constraints on the oxygen uptake rate and ATP synthase (“Methods”), we set the upper bounds on the oxygen uptake rate and ATP synthase to 40 mmol/gDCW/h and 70 mmol/gDCW/h, respectively. The glucose uptake rate was left unconstrained.

In agreement with the experiments, the model predicted that the minimal glucose uptake of 7.51 mmol/gDCW/h is required to attain the specific growth rate of 0.73 1/h when the lower bound of the flux through ATP hydrolysis is set to 0 mmol/gDCW/h (Fig. 5c, d). Also consistent with the experiments, with the increase of the minimally required ATP hydrolysis flux, the required minimal glucose uptake was increasing (Fig. 5d) simultaneously with an increase of the ATP synthesis flux and minimal oxygen uptake (Fig. 5e, f), while the specific growth rate remained stable (Fig. 5c). For the ATP hydrolysis flux of 37 mmol/gDCW/h, the minimal glucose uptake was 9.56 mmol/gDCW/h and the slope of the minimal glucose and oxygen uptake became steeper (Fig. 5d, f). When the ATP hydrolysis flux reached 44 mmol/gDCW/h, the oxygen uptake rate and ATP synthase flux simultaneously attained their upper bounds (Fig. 5e, f). The corresponding minimal glucose uptake was 11.89 mmol/gDCW/h, which was consistent with Ebert et al. [7] (11.6 ± 1.2 mmol/gDCW/h). After this point, the required minimal glucose uptake started to decline (Fig. 5d) together with a decline in the specific growth rate (Fig. 5c). For the ATP hydrolysis flux of 73 mmol/gDCW/h, the model predicted the specific growth rate of 0.25 1/h and the minimal glucose uptake rate of 8.54 mmol/gDCW/h, which was slightly more than what was reported in the Ebert et al. [7] (7.5 ± 0.8 mmol/gDCW/h).

The thermodynamically curated core stoichiometric model described well the qualitative behavior of *P. putida* in the stress condition of increased ATP demand. However, the model failed to capture a decrease of the specific growth rate for DNP concentrations in the range of 300–700 mg/l (Fig. 5c). Possible explanations for this discrepancy are that the decrease of specific growth rate in this region might be due to: (i) kinetic effects that cannot be captured by stoichiometric models; (ii) the intrinsic toxicity of DNP, which was not modeled. It is also important to observe that in Ebert et al. [7] the increased ATP demand was indirectly induced by tittering different levels of DNP, whereas we simulated that effect by increasing the ATP hydrolysis flux. Since *P. putida* does not necessarily respond to a linear increase in the DNP levels by linearly increasing the ATP hydrolysis, the exact correspondence of the data points in the graphs obtained through experiments and computational simulation was not expected.

#### Improving the robustness of *P. putida* under stress conditions

We devised a metabolic engineering strategy that will allow *P. putida* to maintain the specific growth rate for more severe stress conditions. To this end, we computed the steady-state metabolic flux and metabolite concentration vectors for the ATP hydrolysis flux of 44 mmol/gDCW/h. We then built a population of 50,000 kinetic models around the computed steady-state, and computed the control coefficients for all fluxes and concentrations in the metabolic network.

**Fig. 6:**
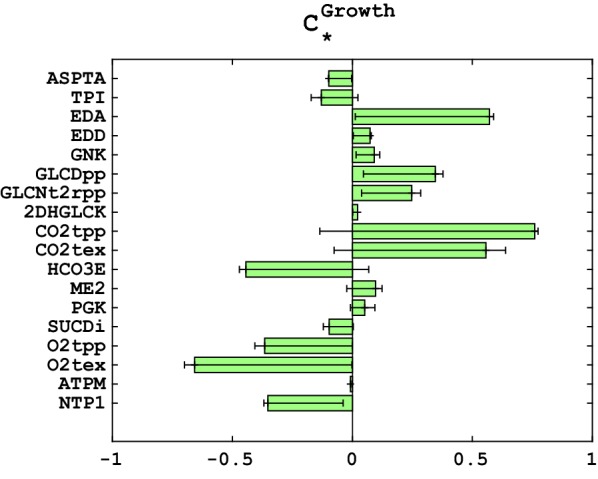
Control coefficients of the specific growth rate in the stress conditions. The green bars are the mean values of the control coefficients, whereas the error bars correspond to the 25 and 75 percentiles of the distributions

Analysis of the control coefficients for the specific growth rate revealed several strategies for maintaining high growth in the presence of stress agent 2,4-dinitrophenol that increases ATP demand (Fig. 6). The major positive control over the specific growth at this stress condition have the key enzymes from the Entner–Doudoroff pathway (EDA, EDD and GNK), e.g., the twofold increase in activity of EDA would improve the specific growth by more than 50%. That is, the extra ATP demand is balanced with higher glucose uptake and glucose catabolism through ED pathway (Additional file 2: Figure S4, Additional file 3: Figure S5 and Additional file 4: Figure S6). Furthermore, these enzymes have a positive control over NADPH production (Additional file 5: Figure S7), which is necessary to fuel proton-motive-force-driven efflux pumps, the major mechanism of solvent tolerance in *P. putida* [95] or to reduce stress through antioxidant systems that utilize NADPH [96].

Similarly, our analysis suggests that an increase in the activity of GLCDpp that catalyzes the conversion of glucose to periplasmic gluconate would increase the specific growth, i.e., the twofold increase in GLCDpp activity would result in improved specific growth by ~ 40% (Fig. 6). The twofold decrease in the activity of triose-phosphate isomerase (TPI) would result in a 13% increase in the specific growth. Furthermore, the twofold decrease in the activity of aspartate transaminase (ASPTA) and succinate dehydrogenase (SUCDi) would also increase the specific growth by 9.5% and 9.9%, respectively. The reason for these effects is coupling through redox, protons, and electrons, which is in part stoichiometric. However, if one observes closer the mass balances for redox, protons, and electrons, they allow more flexibility in the coupled reactions as opposite to main carbon balances. This result further demonstrates the values of kinetic models, which for a given set of kinetic parameters can unambiguously the responses to genetic and environmental perturbations.

Interestingly, our results also show a positive control of malic enzyme (ME2) over the specific growth. Together with pyruvate carboxylase (PC), ME2 forms the pyruvate shunt that in *P. putida* grown on glucose channels malate to oxaloacetate [63, 97]. Since PC hydrolyzes ATP and ME2 produces NADPH, the pyruvate shunt is considered to be energetically costly, thus affecting cellular growth, but potentially useful for the redox metabolism [97]. However, in the studied stress condition, the flux control coefficients show that ME2 activity increase results in an increase of the flux through PC (Additional file 6: Figure S8) but impacts also other fluxes in the metabolic network, including the remaining reactions related to the ATP metabolism (43 reactions from the core network and the majority of the lumped reactions). Additionally, ME2 activity increase causes increase in the glucose uptake, and GLCDpp and EDA/EDD fluxes (Additional file 2: Figure S4, Additional file 3: Figure S5, Additional file 4: Figure S6 and Additional file 7: Figure S9), which results in increased ATP production and specific growth (Additional file 8: Figure S10 and Fig. 6). The overall positive effects of increased ME2 activity on ATP production and growth outweigh the negative effect of ATP hydrolysis by PC. Without large-scale kinetic models it would be difficult to uncover such complex and unintuitive interactions in the metabolic network.

We found a strong correlation between the control coefficients of the specific growth and the concentration control coefficients of the cytosolic ATP (Additional file 1: Table S6). Indeed, the Pearson coefficient was 0.8 between these two sets of control coefficients with respect to their top controlling enzymes. Moreover, the top enzymes had a consistent control over the specific growth and the cytosolic ATP concentration. That is, the enzymes that had a positive control over the specific growth had a positive control over the cytosolic ATP concentration, and similarly for the enzymes with a negative control. This indicated that the key factor for improving the specific growth under severe stress conditions is to improve the ATP production in cytosol.

## Conclusions

This study presents the first thermodynamically curated genome-scale model of *P. putida*. Thermodynamic curation makes the curated GEM iJN1411 amenable for integrating metabolomics data. The integration of thermodynamics data into models restricts the available flux and concentration spaces [35, 39] because thermodynamics determines the directionality in which reactions can operate [35, 37]. For example, Flux Balance Analysis (FBA) performed on iJN1411 indicated that 108 reactions could operate in both forward and reverse direction (bi-directional reactions) while still being consistent with the integrated fluxomics data [63]. However, when additional metabolomics data [64] were integrated with TFA, 21 out of these 108 reactions could not operate in both directions due to thermodynamic constraints (Additional file 1: Table S4). The thermodynamically curated iJN1411 was further used to develop a family of three systematically reduced models of *P. putida* central carbon metabolism that lend themselves for a wide gamut of metabolic engineering studies.

Current metabolomics measurement techniques do not allow for distinguishing concentrations of the same species in different compartments. Consequently, when integrating metabolomics data in constraint-based techniques that consider thermodynamics such as the energy balance analysis [98], the network-embedded thermodynamic analysis [99] and the thermodynamics-based flux analysis [35, 36, 38, 39], it is commonly assumed that the concentrations of a metabolite appearing in several compartments are identical and constrained within experimentally measured values. We proposed here a novel set of constraints within TFA that enable integration of metabolomics data without imposing this restrictive assumption. In this formulation, we model concentrations of metabolites that exist in several compartments as distinct quantities, and, at the same time, we preserve the consistency of their values with experimentally measured values for the whole cell. This way, we ensure that the set of possible metabolic outcomes predicted by the model encompasses the actual cellular physiology.

Finally, we derived here the kinetic models of *P. putida*’s central carbon metabolism containing 775 reactions and 245 metabolites that comprise pathways from glycolysis and gluconeogenesis, pentose phosphate pathway, pyruvate metabolism, TCA cycle, and oxidative phosphorylation. Considering their size, scope, and level of details, the derived models are the largest kinetic model of this organism available in the literature to this date. The potential applications of the developed kinetic models were illustrated in two studies of *P. putida* metabolism.

## Methods

### Considering cellular compartments while integrating metabolomics data

Here we propose a novel set of constraints that allow for considering concentrations of the same species across different compartments while maintaining the consistency with the experimental measurements.

For the concentration $C_{M}$ of a metabolite *M* measured in the range $C_{M} \in \left( {\underline{{C_{M} }} ,\overline{{C_{M} }} } \right)$ we have:

1 $$C_{M} = \frac{{N_{t} }}{{V_{t} }} = \frac{{\mathop \sum \nolimits_{i} N_{Ci} }}{{\mathop \sum \nolimits_{i} V_{Ci} }},$$

where $N_{t}$ is the number of moles of *M* and $V_{t}$ is the total volume of the cell. $N_{Ci}$ and $V_{Ci}$ are the corresponding quantities in compartments *i*. Considering that $\mathop \sum \nolimits_{i} V_{Ci} = V_{t}$, i.e., $\mathop \sum \nolimits_{i} \frac{{V_{Ci} }}{Vt} = \mathop \sum \nolimits_{i} \alpha_{i} = 1$, by dividing (1) with $V_{t}$ we obtain

2 $$C_{M} = \frac{{\mathop \sum \nolimits_{i} \frac{{N_{Ci} }}{{V_{t} }}\frac{{V_{Ci} }}{{V_{Ci} }}}}{{\mathop \sum \nolimits_{i} \frac{{V_{Ci} }}{Vt}}} = \frac{{\mathop \sum \nolimits_{i} \alpha_{i} C_{Mi} }}{{\mathop \sum \nolimits_{i} \alpha_{i} }},$$

where $C_{Mi}$ is the concentration of metabolite *M* in the compartment *i* and $\alpha_{i}$ is the volume fraction of the compartment *i* with respect to the entire cell. Observe that $\alpha_{i}$ and $C_{Mi}$ are positive quantities.

If we apply logarithm to (2), we have:

3 $$\log C_{M} = \log \frac{{\mathop \sum \nolimits_{i} \alpha_{i} C_{Mi} }}{{\mathop \sum \nolimits_{i} \alpha_{i} }}.$$

Considering that log is a concave function, we can use Jensen’s inequality [100] where for a concave function $\varphi$ and positive weights $\alpha_{i}$ it holds that:

4 $$\varphi \left( {\frac{{\mathop \sum \nolimits_{i} \alpha_{i} x_{i} }}{{\mathop \sum \nolimits_{i} \alpha_{i} }}} \right) \ge \frac{{\mathop \sum \nolimits_{i} \alpha_{i} \varphi \left( {x_{i} } \right)}}{{\mathop \sum \nolimits_{i} \alpha_{i} }}.$$

Therefore, by combining (3), (4) we get:

5 $$\log C_{M} = \log \frac{{\mathop \sum \nolimits_{i} \alpha_{i} C_{Mi} }}{{\mathop \sum \nolimits_{i} \alpha_{i} }} \ge \sum \alpha_{i} \log C_{Mi} .$$

Moreover, if we denote the physiological lower and upper bound on intracellular metabolite concentrations as LB= 1 μM and UB=50 mM, respectively, then the upper bound on $C_{Mi}$, $\overline{{C_{Mi} }}$, can be derived from the following expression:

6 $$\overline{{C_{M} }} = \alpha_{i} \overline{{C_{Mi} }} + \left( {1 - \alpha_{i} } \right)*{\text{LB}},$$

hence

7 $$\overline{{C_{Mi} }} = \frac{{\overline{{C_{M} }} - \left( {1 - \alpha_{i} } \right)*{\text{LB}}}}{{\alpha_{i} }}.$$

To prevent the case $\overline{{C_{Mi} }} > {\text{UB}}$ for some values of $\alpha_{i}$, we set the upper bound on $\overline{{C_{Mi} }}$ as follows:

8 $$\overline{{C_{Mi} }} = \hbox{min} \left( {\frac{{\overline{{C_{M} }} - \left( {1 - \alpha_{i} } \right)*{\text{LB}}}}{{\alpha_{i} }}, {\text{UB}}} \right).$$

Analogously for the lower bound on the concentration of the metabolite *M* in the compartment *i*, $\underline{{C_{Mi} }}$, we have:

9 $$\underline{{C_{Mi} }} = \hbox{max} \left( {\frac{{\underline{{C_{M} }} - \left( {1 - \alpha_{i} } \right)*{\text{UB}}}}{{\alpha_{i} }}, {\text{LB}}} \right).$$

Therefore, instead of using *i* constraints on the compartment species of metabolite *M* in the form of $\log \underline{{C_{M} }} \le \log C_{Mi} \le \log \overline{{C_{M} }}$, we propose to use *i *+ 2 constraints providing more flexibility and relaxing the assumption on equal concentrations of metabolite M in all compartments:

10 $$\log \underline{{C_{Mi} }} \le \log C_{Mi} \le \log \overline{{C_{Mi} }}$$

together with (5) and

11 $$\log \underline{{C_{M} }} \le \log C_{M} \le \log \overline{{C_{M} }} ,$$

where $\underline{{C_{Mi} }}$ and $\overline{{C_{Mi} }}$ are computed as in (8), (9).

The volume fractions of cytosol, $\alpha_{1}$, and periplasm, $\alpha_{2}$, were taken respectively as 0.88 and 0.12 [101].

### Gap-filling of thermodynamically curated iJN1411

In the gap-filling procedure [60], we carried out MILP using the matTFA toolbox [102] to find a minimal set of reactions that should be added to iJN1411 to match experimentally measured values of glucose uptake, specific growth rate, and ATP concentration. The candidate reactions for the gap-filling were taken from iJO1366 GEM of *E. coli*. More precisely, we appended reactions from iJO1366 into iJN1411 to obtain a composite model. We then removed duplicate reactions from the composite model along with phosphofructokinase (PFK) that is experimentally shown to be absent from *P. putida* metabolism [65]. Compared to iJN1411 the composite model had additional 1201 reactions originating from iJO1366. We performed MILP for the imposed task, and we found that it is sufficient to add one out of 1201 reactions (sulfate adenyltransferase (SADT2)) from iJO1366 to iJN1411 to obtain consistency of iJN1411 TFA solutions with the experimental data.

### Systematic reduction of iJN1411

We used the redGEM [76] and lumpGEM [77] algorithms to deliver reduced models of three different sizes (referred in “Results and discussion” section as D1, D2 and D3). The first step in the redGEM algorithm is to select the metabolic subsystems of interest around which the reduced models are built. We selected the following six metabolic subsystems from iJN1411: glycolysis and gluconeogenesis, pentose phosphate pathway, pyruvate metabolism, TCA cycle, and oxidative phosphorylation. From the reactions belonging to these six subsystems, we removed all cofactor pairs and small metabolites such as protons, phosphate groups, and inorganics. We then used a graph search algorithm to identify all one-reaction, two-reaction, and three-reaction steps pairwise connections between six subsystems and formed the core metabolic networks of D1, D2 and D3 model, respectively. We next performed another graph search to find the connections of D1–D3 core networks with the extracellular space. With this step the core networks of D1, D2 and D3 models were finalized.

We then used the lumpGEM [77] algorithm to connect the core networks of D1, D2 and D3 with the building blocks of the iJN1411 biomass reaction. For each of 102 iJN1411 biomass building blocks (BBBs), lumpGEM identified a set of alternative minimal subnetworks that were able to connect precursors belonging to the core network and the BBB. The size of minimal networks is denoted *S*_min_ [77]. For some studies it is of interest to identify subnetwork of higher sizes. Herein, we identified subnetworks of the size *S*_min_ + 2. Finally, lumpGEM collapses the identified subnetworks into lumped reactions, which together with the core networks constitute the core reduced model.

The D1 model consisted of: (i) the D1 core network formed by the reactions and metabolites from the six subsystems and the reactions that belonged to one-reaction-step pairwise connections between these six subsystems [76] (Fig. 1); and (ii) lumped reactions that connected the D1 core network with the BBBs. The D2 model contained: (i) the D2 core network containing the D1 core network and the reactions and metabolites that belonged to two-reaction-step pairwise connections between the six subsystems (Fig. 1); and (ii) lumped reactions that connected the core network of D2 and the BBBs. The reactions that belonged to the two-reaction-step pairwise connections between the subsystems were predominantly from the fatty acid and amino acid metabolism (Additional file 9: File S2). The core network of the highest complexity model, D3, included also the reactions and metabolites from the three-reaction-step pairwise connections between the six subsystems (Fig. 1). The reactions included into the D3 core network were mostly from glyoxylate and dicarboxylate metabolism and folate biosynthesis (Additional file 10: File S3).

### Consistency checks of core reduced models

We performed a battery of tests to validate the consistency of the systemic properties of the core reduced models D1, D2 and D3 with their GEM counterpart, iJN1411. Here we present and discuss results for D2, the results for D1 and D3 are provided in Additional file 11: File S4.

We first performed FBA and TFA for the glucose uptake of 10 mmol/gDCW/h, and we found the identical maximum specific growth rate of *μ* = 0.94 h^−1^ for both D2 and iJN1411, meaning that D2 was able to capture well the physiology of the growth on glucose.

We then carried out the comparison of essential genes between D2 and GEM. In silico gene deletion represents one of the most common analysis of metabolic networks, and it is used to assess the predictive potential of the model [10] or to identify main genetic targets for strain engineering [16, 103]. Out of 314 genes that D2 shared with GEM, we identified 47 as in silico essential. Out of these 47, 36 were essential in both D2 and GEM and 11 were essential in D2 only (Additional file 1: Table S5). These 11 genes were essential in D2 because this model was missing some of the alternative pathways from GEM. For example, aceF PP_0338 (encoding for acetyltransferase component of pyruvate dehydrogenase complex) and aceE PP_0339 (encoding for pyruvate dehydrogenase, E1 component) are essential in D2 because they encode for enzymes necessary for synthesizing acetyl-CoA from pyruvate, whereas GEM contains additional alternative pathways for this synthesis. Interestingly, among the 11 genes is tpiA PP_4715 encoding for triose-phosphate isomerase, which is reported as essential in the literature [78].

We next performed thermodynamic-based variability analysis (TVA) on all common reactions and metabolites of D2 and GEM and compared their thermodynamically allowable ranges. We obtained consistent flux ranges for the majority of the reactions, and 131 reactions were less flexible in D2 than in GEM (Additional file 12: Figure S3). Most of these reactions were in the upper glycolysis such as GAD2ktpp (gluconate 2 dehydrogenase periplasm), GLCDpp (glucose dehydrogenase), HEX 1 (hexokinase) and GNK (gluconokinase), and gluconeogenesis such as PGK (phosphoglycerate kinase), PGM (phosphoglycerate mutase) and ENO (enolase). Additional flexibility of these reactions in GEM comes from the pathways of starch and sucrose metabolism and cell envelope biosynthesis cellulose metabolism, which are absent in D2. The allowable ranges of concentrations of common metabolites of D2 and GEM were consistent. Similar result was reported for the case of *E. coli* where the discrepancy in concentration ranges was reported for only few metabolites [76].

### Preconfiguring stoichiometric model for kinetic studies of wild-type physiology

We expanded the stoichiometric network of D2 by adding the reactions that model free diffusion to extracellular space of all intracellular metabolites that: (i) have less than 10 carbon atoms and do not contain phosphate or CoA; and (ii) do not have an existing transport reaction in the model. This was done to model a possibility that small amounts of these metabolites were produced during fermentation but in insufficient quantities for experimental detection. The expanded model contained 768 reactions and 339 metabolites across cytosol, periplasm, and extracellular space.

Based on the data provided in del Castillo et al. [63], we integrated into the model the experimentally measured rates of glucose uptake and biomass growth and we forced the secretion of d-gluconate and 2-dehydro-d-gluconate by putting a lower bound on their exchange reactions to 0.3 mmol/gDCW/h. For the remaining carbon-based by-products, we allowed only their basal secretion by constraining their transport rates to the extracellular space (10^−6^–10^−3^ mmol/gDCW/h) following the common observation in the literature that *P. putida* can break the carbon down almost without any by-product formation [7]. Furthermore, we integrated 57 experimentally measured intracellular metabolite concentrations [64]. In the model, 12 out of the 57 measured metabolites appear in both cytosol and periplasm. The concentration values of these 12 metabolites were measured per cell and not per compartments, and as discussed previously, to integrate this information for each species in the two compartments only two additional constraints were added in TFA. Overall, these 57 measurements provided constraints for 69 metabolite concentrations in the model.

We then imposed constraints based on several additional assumptions: (i) TCA cycle was complete [7, 78]; (ii) two glutamate dehydrogenases (GLUDx and GLUDy) were operating towards production of l-glutamate; (iii) dihydrolipoamide S-succinyltransferase was generating NADH from NAD+ [104]; (iv) acetaldehyde dehydrogenase (ACALD) was producing acetaldehyde; (v) ribulose 5-phosphate 3-epimerase (RPE) was converting d-ribulose 5-phosphate to d-xylulose 5-phosphate; (vi) adenylate kinase (ADK1) and nucleoside-diphosphate kinase (NDPK1) were consuming ATP; and (viii) GTP-dependent adenylate kinase (ADK3) was consuming GTP.

### Preconfiguring stoichiometric model for kinetic studies of stress conditions

The stoichiometric model was reconfigured in the following way: (i) we constrained the specific growth rate in the range 0.43 ± 0.2 1/h and the glucose uptake in the range 11.6 ± 1.2 mmol/gDCW/h. These values correspond to the concentration of 700 mg/l of DNP in the experimental study or 44 mmol/gDCW/h in the simulation study (Fig. 5d); (ii) the directionalities of 26 reactions from the glycolysis, gluconeogenesis, PPP and TCA were constrained by putting lower and upper bounds from Ebert et al. [7] Interestingly, the reported directionality of TKT2 in this physiological condition was opposite than it was assumed in the study of wild-type physiology; (iii) two glutamate dehydrogenases were operating towards production of l-glutamate; (iv) dihydrolipoamide S-succinyltransferase was operating towards production of NADH from NAD+ [104].

We performed TFA with so configured stoichiometric model, and we found that six reactions (acetaldehyde dehydrogenase acetylating, adenylate kinase, adenylate kinase GTP, sodium proton antiporter, nucleoside diphosphate kinase ATP:GDP and phosphate transport via symport periplasm) could operate in both directions whilst still satisfying the integrated data. To fix the directionalities of these six reactions, we performed another TFA where we minimized the sum of the fluxes in the metabolic network under the constraint that at least 99% of the observed specific growth rate should be attained.

### Sensitivity analysis of metabolic responses to maximal rates in the oxygen uptake and ATP synthesis

Depending on physiological conditions, maximal rates of oxygen uptake and ATP synthase in *P. putida* can take a wide range of values. For instance, in optimally grown *P. putida,* oxygen uptake rate is about 15 mm/gDCW/h [10], while in the stress conditions it can go above 50 mm/gDCW/h [7]. To investigate the effects of the maximal rates on model predictions, we constrained upper bound on biomass growth to 0.73 1/h and we performed multiple TFAs for different combinations of maximal allowed rates of oxygen uptake and ATP synthesis.

**Fig. 7:**
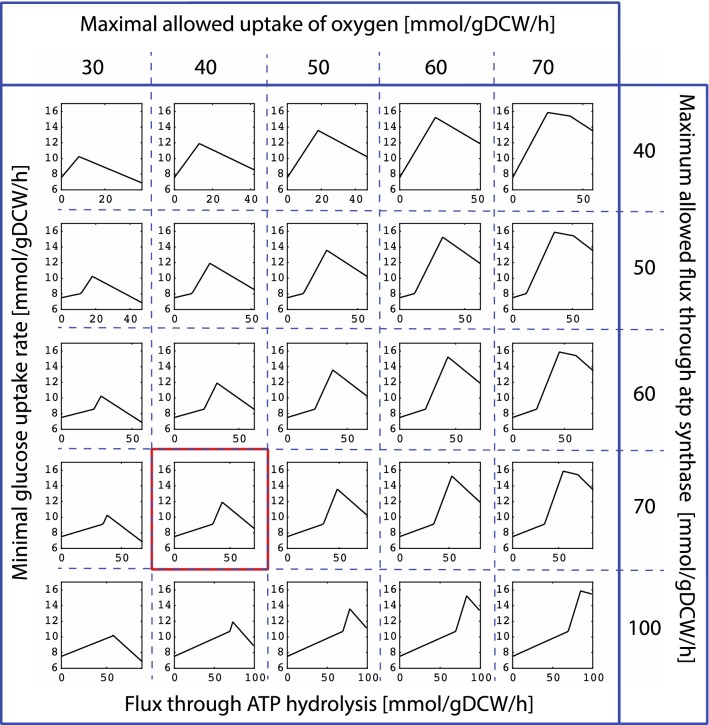
Minimal glucose uptake rate as a function of ATP hydrolysis flux for different combinations of allowed maximal rates of the oxygen uptake and ATP synthesis. The sensitivity analysis indicates that models with the maximal oxygen uptake rate of 40 mmol/gDCW/h and the ATP synthesis rate of 70 mmol/gDCW/h (red box) are providing the best qualitative agreement with the experimental data [7] while maintaining the model flexibility

We varied the allowed maximal oxygen uptake between 30 and 70 mm/gDCW/h (the range between 40 and 60 mm/gDCW/h was reported in [7]), and the allowed maximal flux through ATP synthase between 40 to 100 mm/gDCW/h. For each combination of oxygen uptake/ATP synthase maximal rates, we computed changes of minimal required glucose uptake with the respect to changes in flux through ATP hydrolysis (Fig. 7).

For the allowed maximal oxygen uptake of 30 mmol/gDCW/h, the peak of the minimal glucose uptake rate was at 10.22 mmol/gDCW/h, which is slightly under the value reported in Ebert et al. [7] (11.6 ± 1.2 mmol/gDCW/h) (Fig. 7). For the allowed maximal oxygen uptake of 40 mmol/gDCW/h, the peak of the minimal glucose uptake rate was at 11.89 mmol/gDCW/h which was within the bounds reported in [7], whereas for the allowed maximal oxygen uptake of 50 mmol/gDCW/h, the peak of minimal glucose uptake rate was above the experimental values (13.56 mmol/gDCW/h). Consequently, we used the bound on allowed maximal oxygen uptake rate of 40 mmol/gDCW/h for our kinetic studies.

Interestingly, the constraint on the allowed maximal ATP synthase rate did not have an effect on the magnitude of the peak value of the minimal glucose uptake rate. Instead, it affected the position of the peak with the respect to the ATP hydrolysis flux (Fig. 7). The higher the ATP synthase rate, the higher ATP hydrolysis flux was required to attain the peak value of the minimal glucose uptake. For example, in the case of the allowed maximal oxygen uptake of 30 mmol/gDCW/h, the ATP hydrolysis flux of 9 and 19 mmol/gDCW/h was required to attain the peak of the minimal glucose uptake of 10.22 mmol/gDCW/h for the allowed maximal ATP synthase rates of 40 and 50 mmol/gDCW/h, respectively. Based on these observations and comparison with the experimental data, one can equally consider values of 50, 60 or 70 mmol/gDCW/h for the upper bound on ATP synthase since all three values describe qualitatively well the experimental data [7] (Figs. 5 and 7). We set the upper bound of ATP synthase to 70 mmol/gDCW/h to keep the maximal flexibility in the model.

### Construction of large-scale kinetic models

To construct the kinetic models, we employed the ORACLE framework. In ORACLE, we remove the mass balances for the extracellular metabolites from the stoichiometry because we consider the concentrations of extracellular metabolites as parameters. The mass balances for water and the corresponding transport reactions were also removed. We then assigned a kinetic mechanism to each of the enzyme catalyzed reactions in the model, and we integrated experimental values for 21 Michaelis constants (*K*_m_’s) that we found for the *Pseudomonas* genus in the Brenda database [81–84]. We next employed the Monte Carlo sampling technique to compute the saturation states of enzymes, and these quantities were used to back-calculate the unknown values of Michaels constants (*K*_m_’s) [41, 43, 45].

The details about this framework are discussed elsewhere [34, 41–50].

## Supplementary information

**Additional file 1: Table S1.** Steady state solution used in building kinetic model. **Table S2.**Comparison of experimentally observed and in silico obtained growth rates. **Table S3.** Abbreviations from Fig. 4. **Table S4.** Differences in directionalities between FBA and TFA. **Table S5.** Gene essentiality in rGEM (D2) and GEM. **Table S6.** Correlation between control coefficients of specific growth and ATP concentration with respect to most important enzymes. **Table S7.** Content of glucose minimal media. **Table S8.** Values of Michaelis constants for the Pseudomonas genus acquired from the Brenda database. **Table S9.** Allowable thermodynamically feasible concentrations of ATP with and without including SADT2 in the metabolic network of *P. putida* and *E. coli* together with the available experimental values.

**Additional file 2: Figure S4.** Distribution of the control coefficients of glucose uptake (GLCtex) with respect to most important enzymes in the stress conditions.

**Additional file 3: Figure S5.** Distribution of the control coeicients of 2-dehydro-3-deoxy-phosphogluconate aldolase (EDA) with respect to most important enzymes in the stress conditions.

**Additional file 4: Figure S6.** Distribution of the control coefficients of 6-phosphogluconate dehydratase (EDD) with respect to most important enzymes in the stress conditions.

**Additional file 5: Figure S7.** Distribution of the control coefficients of cytosolic NADPH with respect to most important enzymes in the stress conditions.

**Additional file 6: Figure S8.** Distribution of the control coefficients of pyruvate carboxylase (PC) with respect to most important enzymes in the stress conditions.

**Additional file 7: Figure S9.** Distribution of the control coefficients of glucose dehydrogenase (GLCDpp) with respect to most important enzymes in the stress conditions.

**Additional file 8: Figure S10.** Distribution of the control coefficients of cytosolic ATP with respect to most important enzymes in the stress conditions.

**Additional file 9: File S2.** List of reactions and metabolites of D2 reduced model.

**Additional file 10: File S3.** List of reactions and metabolites of D3 reduced model.

**Additional file 11: File S4.** Consistency checks for the D1, D2, and D3 reduced models.

**Additional file 12: Figure S3.** Thermodynamic-based variability analysis (TVA) on reactions from glycolysis, gluconeogenesis, pentose phosphate pathway and citric acid cycle of D2 (red) and GEM (black).

**Additional file 13: File S1.** List of reactions and metabolites of D1 reduced model.

## Data Availability

Authors can confirm that all relevant data are included in the article and/or its additional information files.

## Abbreviations

ORACLE: Optimization and Risk Analysis of Complex Living Entities
TFA: Thermodynamics-based Flux Balance Analysis
GEM: GEnome-scale Model
MCA: Metabolic control analysis
iSCHRUNK: in Silico Approach to CHaracterization and Reduction of UNcertainty in the Kinetic Models of Genome-scale Metabolic Networks
