# Supplementary figures and images for: Large-scale kinetic metabolic models of Pseudomonas putida KT2440 for consistent design of metabolic engineering strategies
Source: Biotechnol Biofuels. 2020 Feb 28;13:33. doi: 10.1186/s13068-020-1665-7 (PMC7048048; doi:10.1186/s13068-020-1665-7)

$C_{*}^{GLCtex}$

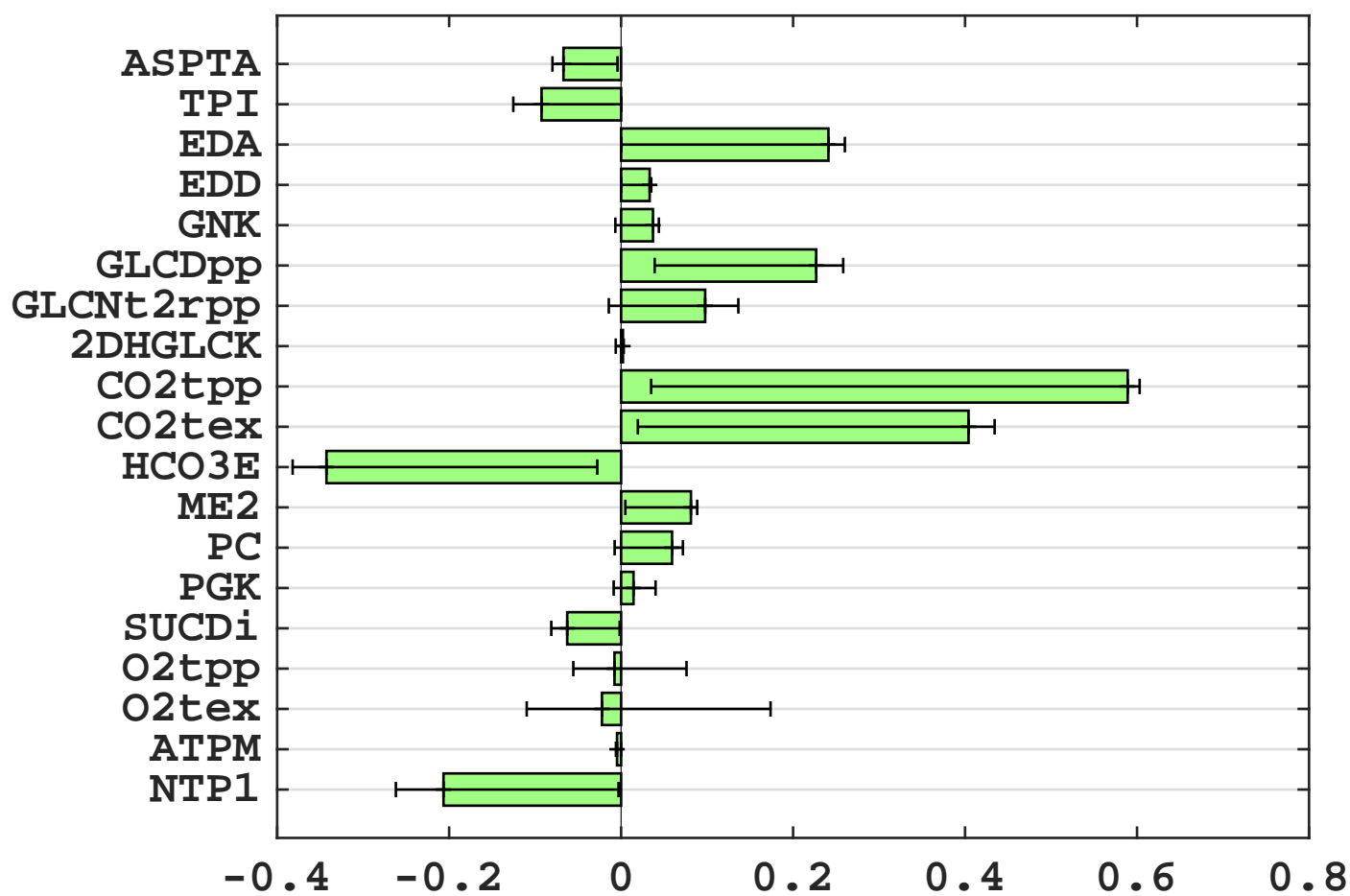

Supplement: Supplementary file 2 — Additional file 2: Figure S4. Distribution of the control coefficients of glucose uptake (GLCtex) with respect to most important enzymes in the stress conditions. [file 13068_2020_1665_MOESM2_ESM.pdf]

$C_*^{\text{EDA}}$

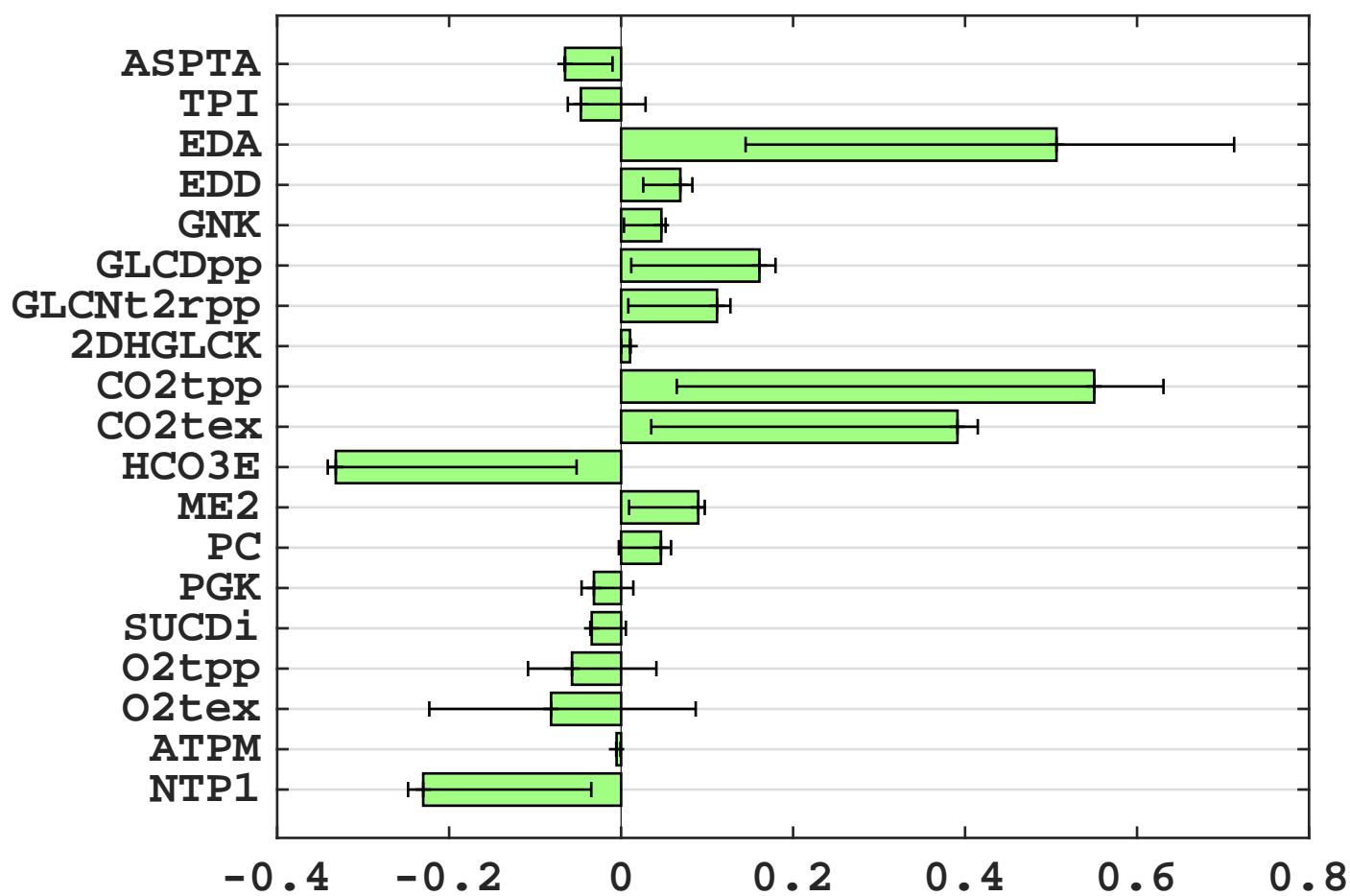

Supplement: Supplementary file 3 — Additional file 3: Figure S5. Distribution of the control coeicients of 2-dehydro-3-deoxy-phosphogluconate aldolase (EDA) with respect to most important enzymes in the stress conditions. [file 13068_2020_1665_MOESM3_ESM.pdf]

$C_*^{\text{EDD}}$

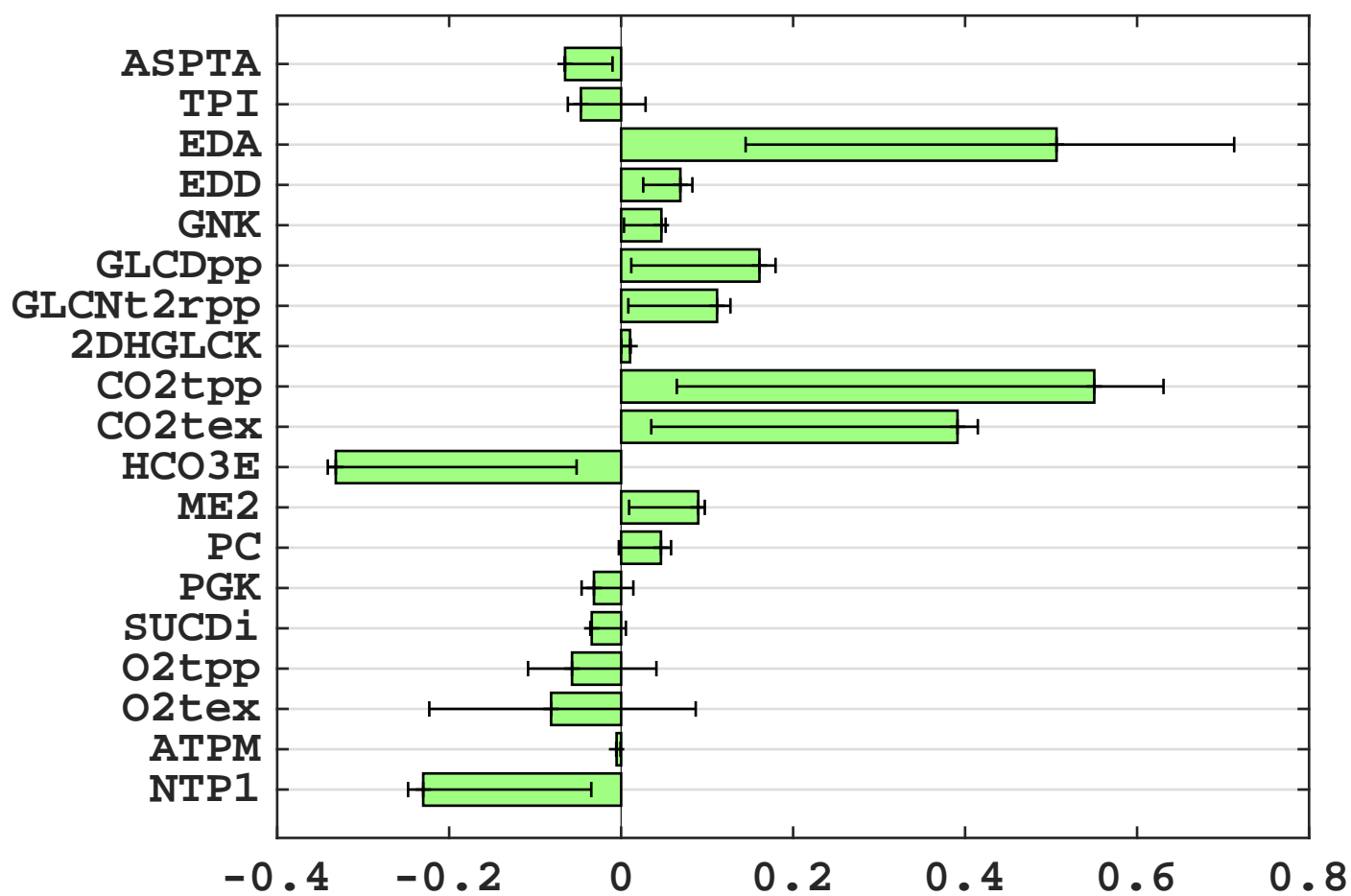

Supplement: Supplementary file 4 — Additional file 4: Figure S6. Distribution of the control coefficients of 6-phosphogluconate dehydratase (EDD) with respect to most important enzymes in the stress conditions. [file 13068_2020_1665_MOESM4_ESM.pdf]

$C_{*}^{\text{nadph}_c}$

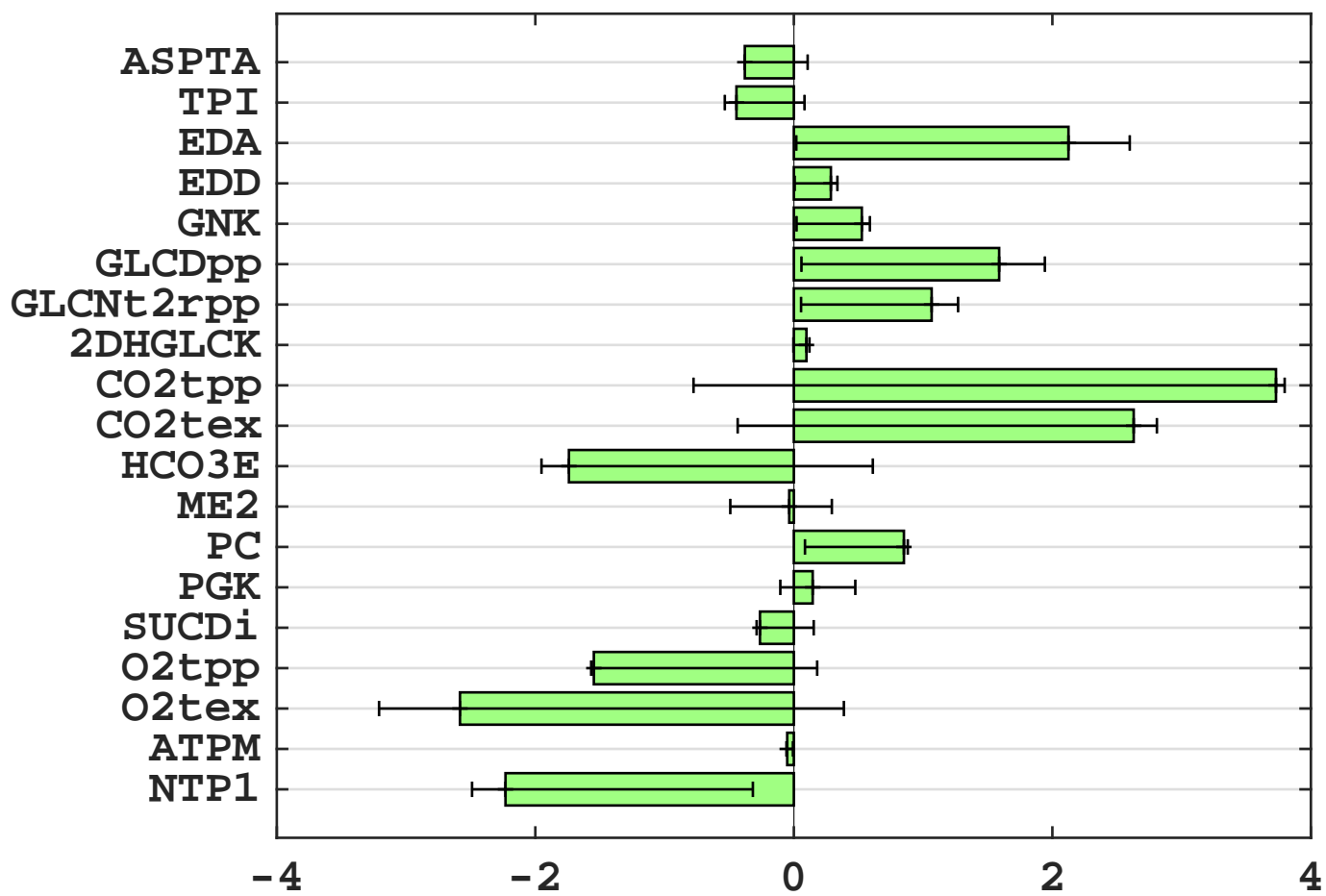

Supplement: Supplementary file 5 — Additional file 5: Figure S7. Distribution of the control coefficients of cytosolic NADPH with respect to most important enzymes in the stress conditions. [file 13068_2020_1665_MOESM5_ESM.pdf]

$C_{*}^{PC}$

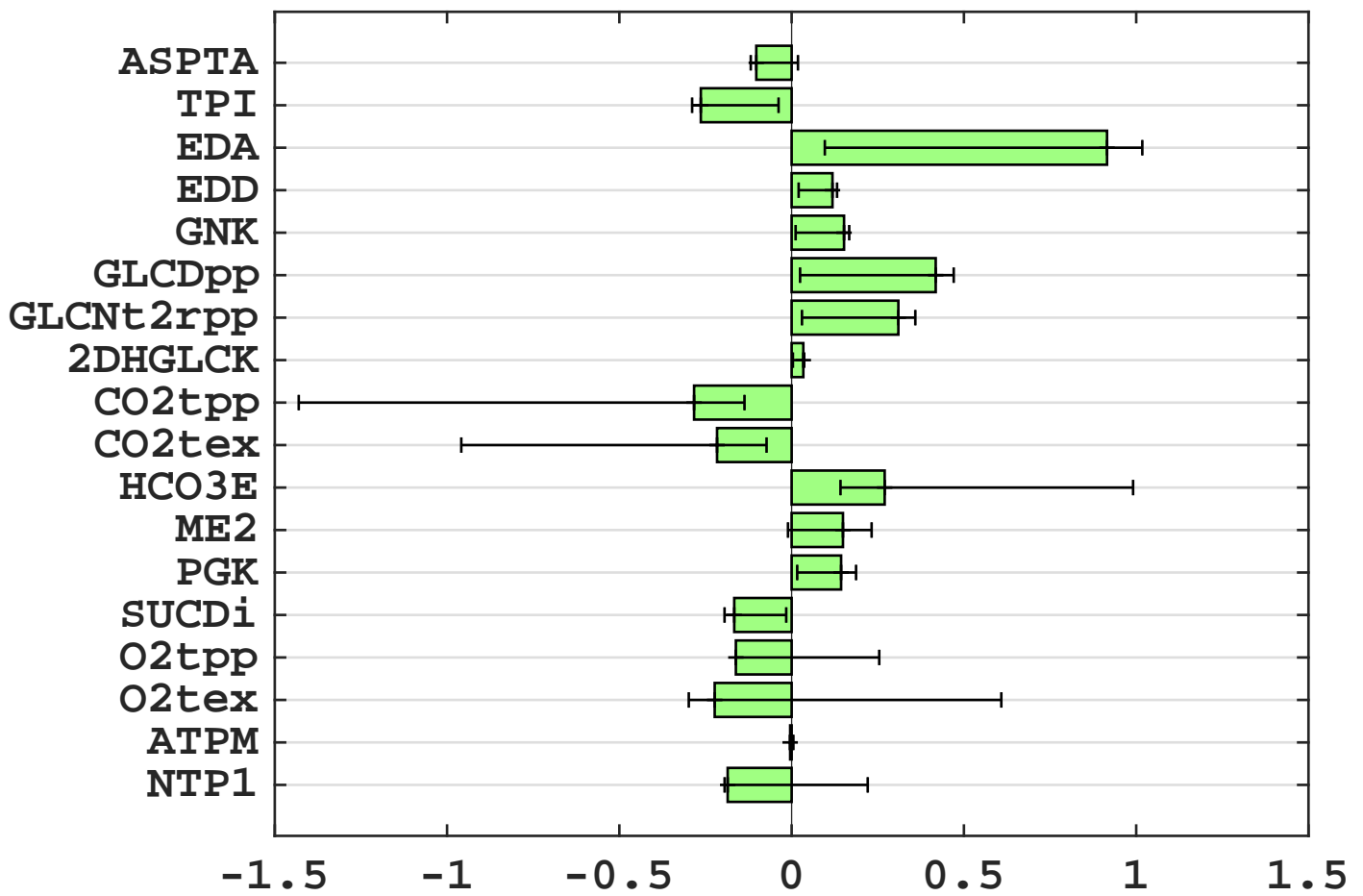

Supplement: Supplementary file 6 — Additional file 6: Figure S8. Distribution of the control coefficients of pyruvate carboxylase (PC) with respect to most important enzymes in the stress conditions. [file 13068_2020_1665_MOESM6_ESM.pdf]

$C_{*}^{GLCDpp}$

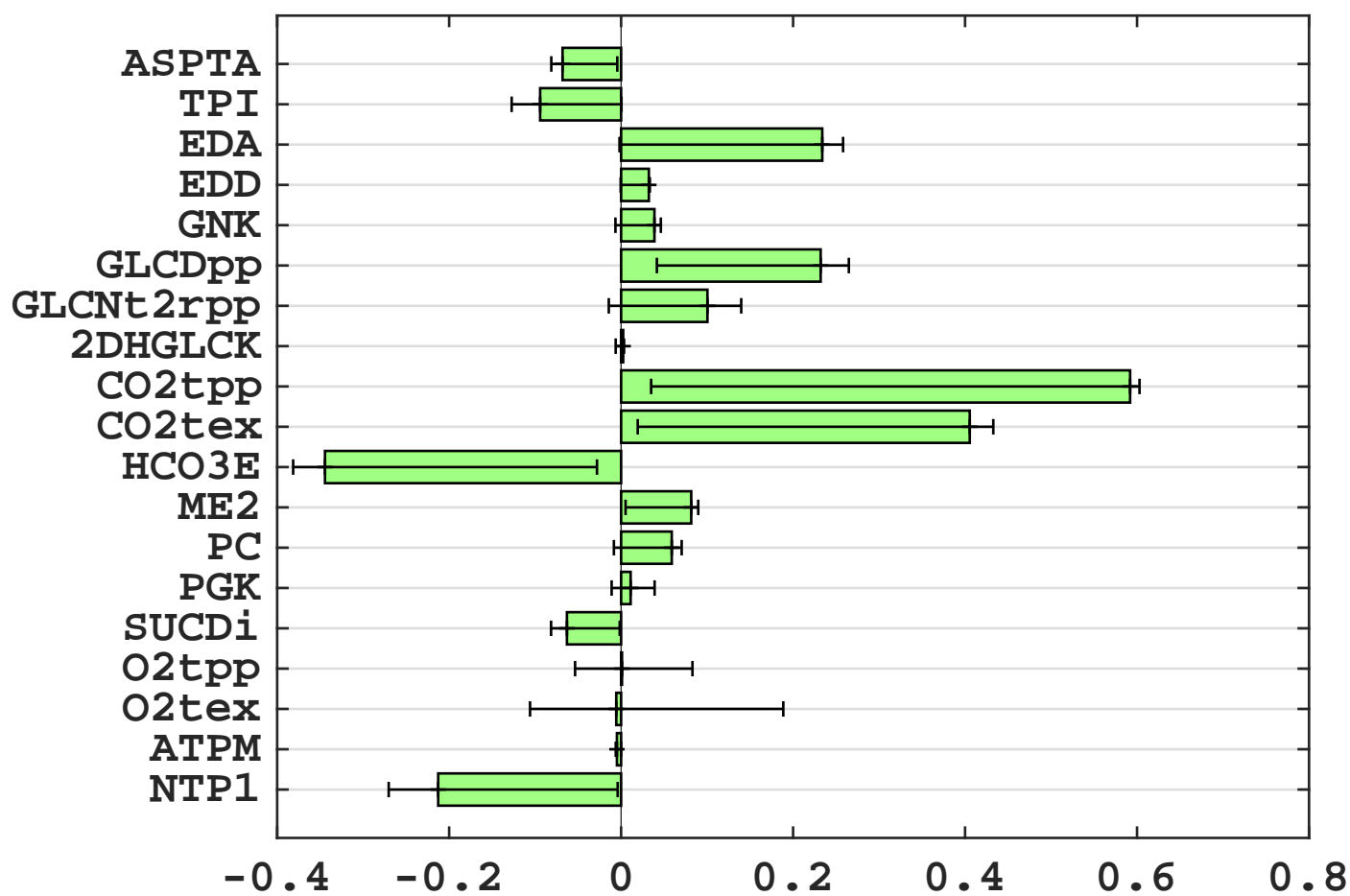

Supplement: Supplementary file 7 — Additional file 7: Figure S9. Distribution of the control coefficients of glucose dehydrogenase (GLCDpp) with respect to most important enzymes in the stress conditions. [file 13068_2020_1665_MOESM7_ESM.pdf]

$$C_{*}^{atp_c}$$

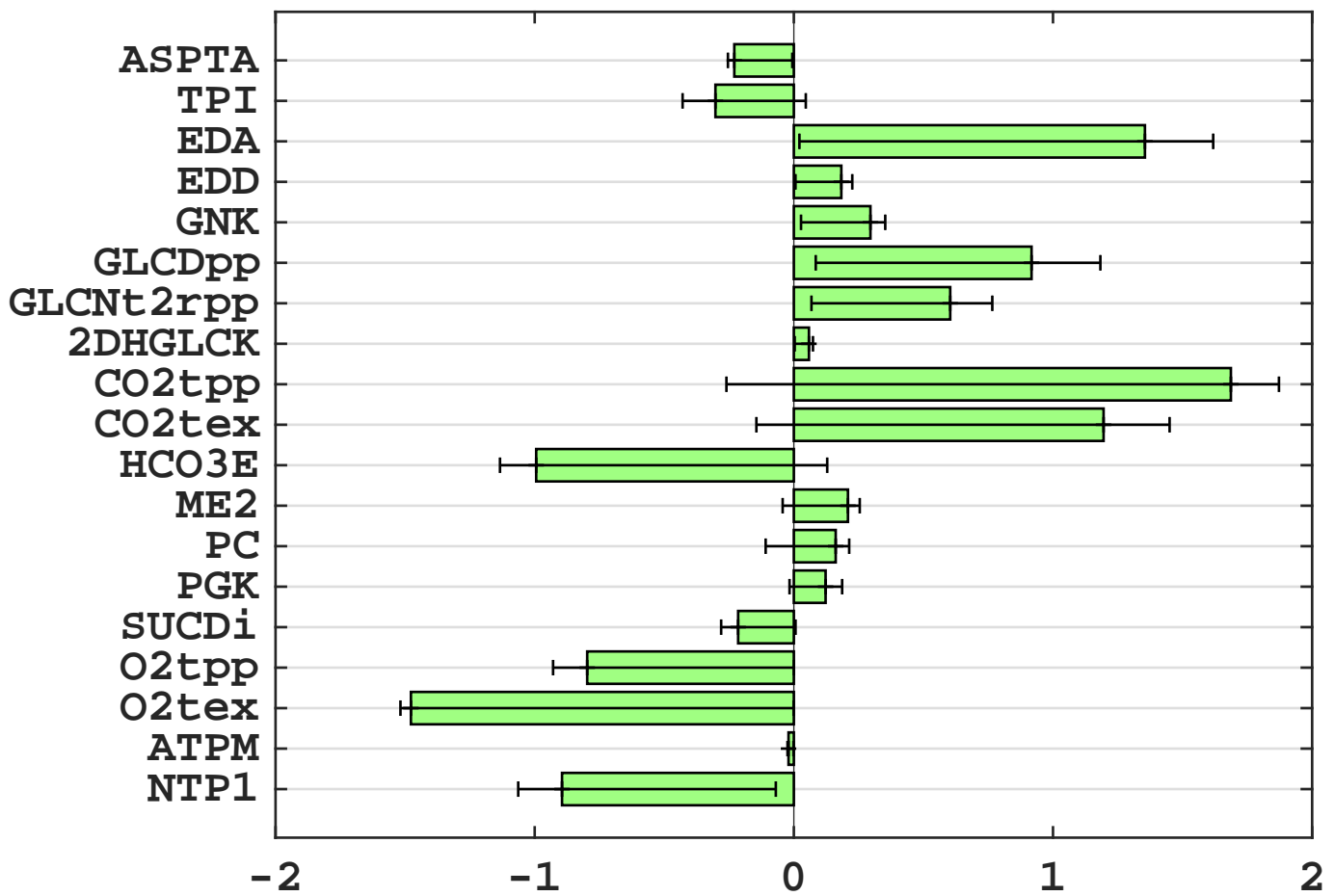

Supplement: Supplementary file 8 — Additional file 8: Figure S10. Distribution of the control coefficients of cytosolic ATP with respect to most important enzymes in the stress conditions. [file 13068_2020_1665_MOESM8_ESM.pdf]

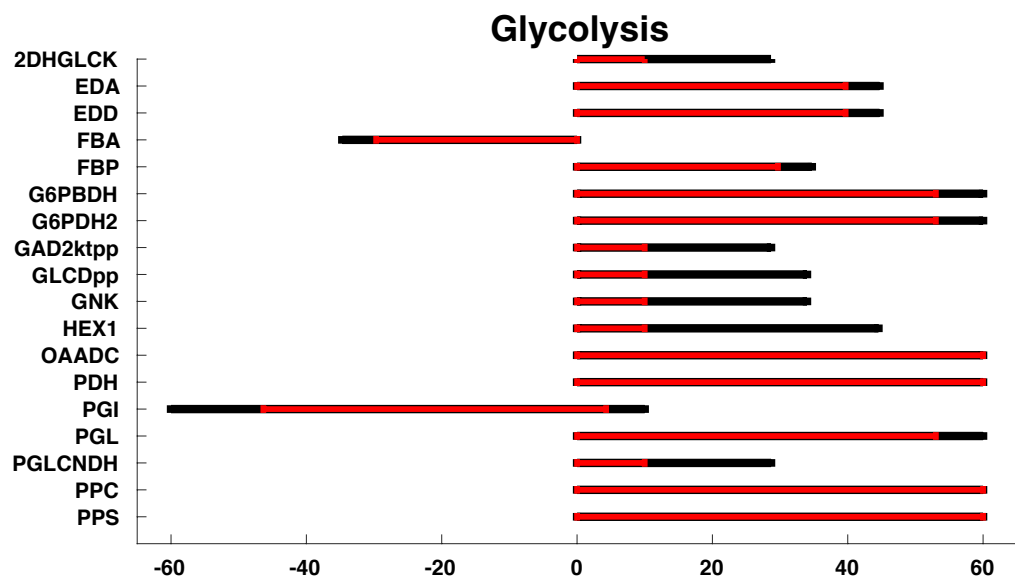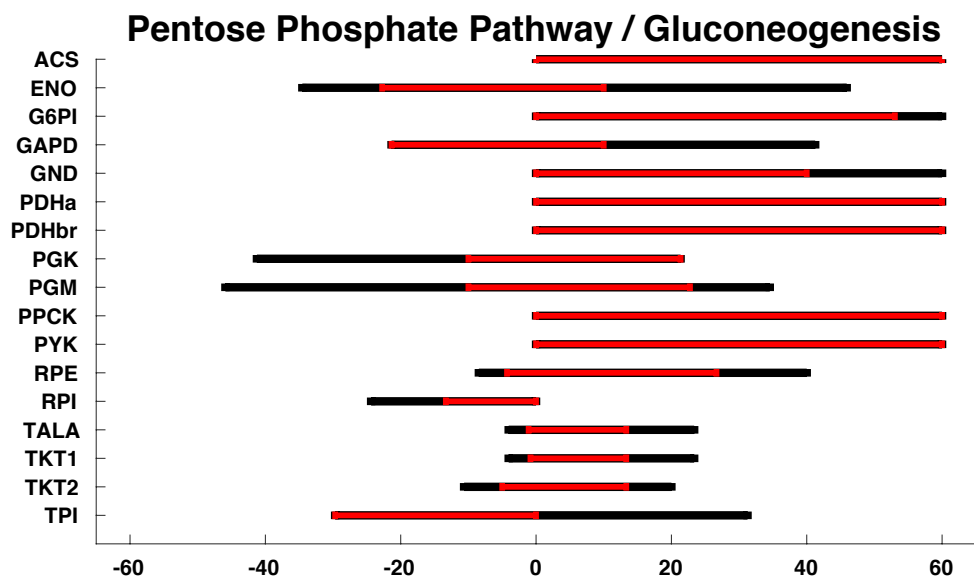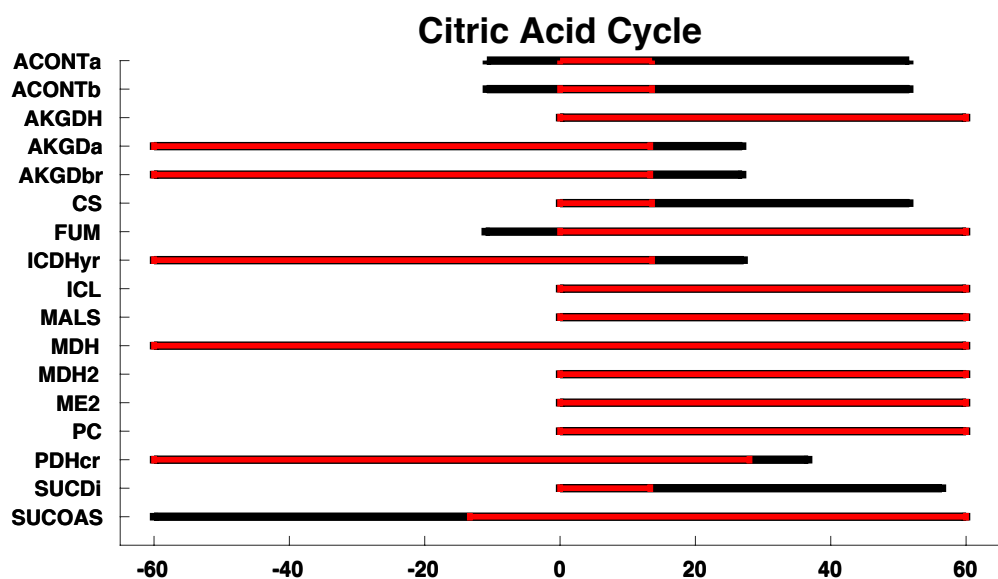

Supplement: Supplementary file 12 — Additional file 12: Figure S3. Thermodynamic-based variability analysis (TVA) on reactions from glycolysis, gluconeogenesis, pentose phosphate pathway and citric acid cycle of D2 (red) and GEM (black). [file 13068_2020_1665_MOESM12_ESM.pdf]
